# A method to improve fishing selectivity through age targeted fishing using life stage distribution modelling

**DOI:** 10.1371/journal.pone.0214459

**Published:** 2019-04-02

**Authors:** Neil M. Burns, David M. Bailey, Peter J. Wright

**Affiliations:** 1 Institute of Biodiversity, Animal Health and Comparative Medicine, University of Glasgow, Glasgow, United Kingdom; 2 Marine Scotland Science, Marine Laboratory, Aberdeen, United Kingdom; Universidad de Cádiz, Facultad de Ciencias del Mar y Ambientales, SPAIN

## Abstract

Understanding spatial distributions of fish species is important to those seeking to manage fisheries and advise on marine developments. Distribution patterns, habitat use, and aggregative behaviour often vary throughout the life cycle and can increase the vulnerability of certain life stages to anthropogenic impacts. Here we investigate distribution changes during the life cycle of whiting (*Merlangius merlangus*) to the west of the UK. Density distributions for age-0, age-1 and mature fish were modelled as functions of environmental variables using generalised additive mixed effects models. The greatest densities of age-0 whiting occurred over finer sediments where temperatures were between 12 to 13°C. Age-0 whiting densities decreased with increasing depth. Higher densities of age-1 whiting were also associated with fine sediments and peaked at 60 m, but this influence was also dependent on proximity to shore. Mature fish, while showing no association with any particular sediment type, were strongly associated with depths >60 m. Geostatistical aggregation curves were used to classify space use and showed persistent aggregations of age-0 whiting occupying inshore waters while age-1 and mature fish were more dispersed and differed among years. The differences in distributions among life stages suggested a general coastal to offshore shift as cohorts developed with mature whiting mainly occupying deep offshore waters. The spatial dynamics and areas of persistent life stage aggregation identified here could enable informed targeting and avoidance of specific age-class whiting to aid bycatch reduction. Given that landing obligation legislation is counterproductive unless it encourages greater fishing selectivity, the ability to avoid this species and undersized individuals would aid conservation measures and fishermen alike.

## Introduction

Understanding the spatial distribution of species remains a long-term goal for ecology and conservation [1]. Recent work has highlighted the importance of knowing the spatial structure of populations to improve their management [2] and aid the recovery of exploited ecosystems [3].The life stages of some fish species are extremely vulnerable to anthropogenic impacts because they persistently occupy particular geographic areas and habitats. Consequently, there is the potential for major and long-term impacts on numerous fish species from anthropogenic activity. Identifying geographic locations important to specific life stages is therefore vital to develop effective marine management strategies [4–6].

Aggregations of spawning adults have received considerable management and research focus owing to their high catchability and vulnerability to fishing [7]. For example, human impacts on some target species like haddock (*Melanogrammus aeglefinus*) have been reduced by protecting geographic areas where spawning is concentrated [7,8]. However, spatial management may also be important where catchability is equally high or higher at other times in the life cycle [9,10]. For instance, the high fishing mortality experienced by juvenile North Sea herring (*Clupea harengus*) is likely to have resulted in recruitment failures in the mid-1970s [11,12]. Indeed, the protection of juvenile aggregations has received widespread support as it can reduce mortality on fish below the legal landing size that are often just a bycatch [13,14].

Several marine fish species show ontogenetic shifts in space use and occupancy patterns. Importantly, localised density patterns may vary over the life cycle and thus increase the vulnerability of certain life stages to anthropogenic activity. Aligning with current legislation [15–17], identifying and managing important geographic areas within regional seas, during the life cycle thus allows protection of essential fish habitat and reduction of anthropogenic impacts during spawning seasons.

Modelling the spatial distribution of fish abundance is possible when abundances are influenced by ecologically relevant, quantifiable abiotic variables [18]. Models can then be used to predict abundances from unsampled sites (model-based interpolation) if interpolations are made in the same time-frame and environmental envelope as the original sampling sites. However, geographic patterns in model residuals can indicate the absence of relevant abiotic variables or the importance of biotic influences like connectivity, dispersal and other biological interactions [19,20]. For example, biotic effects like competition and mutualism will make specific habitats less or more attractive depending on the number of individuals already inhabiting the area.

Distribution models predict abundances based on the number (or presence-absence) of individuals occupying specific habitat types. Therefore, different parameter estimates would be expected depending how close the population is to its carrying capacity [21]. Nevertheless, many distribution models are fitted with abiotic explanatory variables alone and few account directly for biological interactions [22]. Alternative models using density-dependent spatial dynamics can be used to explain large-scale patterns of habitat occupancy [23]. However, Shepherd and Litvak [24] note that spatial variations in characteristics like length-at-age, condition and measures of fitness like reproductive success commonly seen in many fish species [25–27], are incompatible with density-dependence exclusively driving distributions. While variability in parameter estimates owing to biotic influences may have serious implications for extrapolation from models, the consequences for model-based interpolation (given the same time frame and environmental envelope) are less critical [18].

Predicted distributions derived from models can be used to explore the underlying nature of space use. Geostatistical aggregation curves [28] provide a useful tool to describe the relationship between abundance and area occupied and can allow informed classification of space use to be derived. Geostatistical aggregation curves can be constructed from the cumulative proportion of fish abundance per unit area as a function of the proportion of the total area occupied. High density areas display little increase in the total area occupied, whereas more dispersed space use occupies, proportionally, more of the total area. Examining specific life stages allows the varying environmental requirements throughout the life cycle to be described.

Waters to the west of the UK are important to the UK and European fishing industry and contain a wide range of species targeted by demersal trawls including: cod (*Gadus morhua*), haddock, whiting (*Merlangius merlangus*) and *Nephrops* (*Nephrops norvegicus*). Spawning stock biomass and recruitment in cod, haddock and whiting remain much lower that historic levels [29]. A Total Allowable Catch (TAC) has been set at zero since 2006 for whiting [30]. However, management measures, like implementing TACs, effort restrictions and introducing larger net mesh sizes to alleviate pressure on these stocks, have not had the desired effect to the west of the UK. West coast stocks of cod, haddock and whiting may be unable to sustain even the controlled directed fishing pressure they currently experience [31–33].

Fishing within sustainable limits should allow stocks to recover. However, to the west of the UK since the 1960s the *Nephrops* fishery has been of increasing importance and while not directly targeted by the *Nephrops* fishery, many demersal species still experience increased mortality rates as bycatch. Juvenile whiting in particular have been found to constitute a large proportion of this fisheries bycatch [34–37]. Age-0 and age-1 whiting are caught as bycatch by the *Nephrops* fishery and account for 77% of the discards of the juvenile life stage [30]. Direct fishing pressure and the additional pressure from *Nephrops* trawlers catching and discarding whiting and other gadoid species may increase mortality rates enough to suppress overall population growth. Substantially reducing juvenile whiting bycatch has been recommended as essential for the recovery of this stock [38].

Whiting is an important commercial species across the northeast Atlantic [39]. Although the spawning stock biomass is now much reduced, whiting are a major component of the demersal fish biomass west of the UK [35,40]. As a consequence of the low stock size there is currently very little directed fishing for this species off the UK west coast and catches are predominantly discarded. While numbers of later life stages are greatly reduced and recruitment is low [34,35], small, young whiting do persist in inshore waters. For example, following the decline of cod and haddock in the Firth of Clyde, whiting have replaced these other, previously more numerous species, as the major component of gadoid biomass [41]. In contrast to many other gadoids, immature and mature whiting are found together during the spawning season suggesting this species does not exhibit spatially distinct spawning areas [42]. A protracted spawning season from January to June is followed by a pelagic larval phase lasting up to six months and then settlement in inshore waters. After their first winter, juveniles join the adult stock [43,44]. Tagging studies of adults [45] and cohort tracking using otolith microchemistry [46] have shown an ontogenetic trend for offshore dispersal following settlement. However, the distribution of this species throughout its life cycle has yet to be fully quantified. Lacking this knowledge, the implementation of effective management strategies aimed at addressing anthropogenic impacts affecting this species is not possible.

In this study, generalised additive mixed effects models (GAMM) were used to model whiting distribution to the west of Scotland and in the Irish Sea at three life stages from research survey trawls. This allowed the spatio-temporal constancy of aggregation patterns to be examined and persistent areas of importance for the three key life stages to be identified.

## Materials and methods

### Fish life stage abundances

Whiting abundance was calculated from scientific trawl surveys conducted between 2009 and 2015 as part of the Scottish West Coast International Bottom Trawl Survey (SWC-IBTS) and Northern Ireland Ground Fish Survey (NIGFS). The SWC-IBTS survey used a Grand Ouverture Vertical trawl (GOV) towing for a period of 30 minutes. The NIGFS utilises a rock-hopper otter trawl travelling for 1 hour. Catch per unit effort (CPUE) for all tows was standardised to 30 minutes with counts rounded down to the nearest whole fish. Data were downloaded from the ICES DATRAS database [47]. The survey standard protocol is described in the Manual for the International Bottom Trawl Surveys in the Western and Southern Areas [48]. Whiting abundance was calculated for three life stages: age-0, age-1 and mature fish. The abundance of age-0 and age-1 fish was calculated regardless of maturation stage. The abundance of mature whiting was derived from all fish age-1 and above. Surveys conducted between February and March in 2009–2015 provided data on age-1 and mature whiting at the start of the spawning season, while surveys in October and November 2011–2014 provided data on age-0 whiting. In real terms the age difference between age-0 and age-1 whiting is around four months and reflects seasonal changes in abundance.

**Fig 1:**
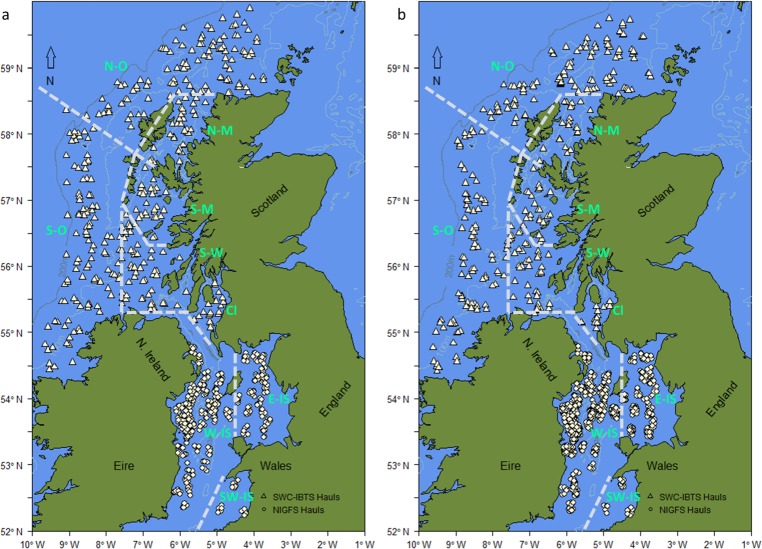
Sample haul locations during the study period (2009–2015). Hauls shown for (a) February-March and (b) October-November surveys. Dotted lines indicate the boundaries defining regions (j) used in the binomial GLMs. Named areas are: (N-O) North Offshore, (S-O) South Offshore, (N-M) North Minch, (S-M) South Minch, (S-W) South West, (Cl) Firth of Clyde, (W-IS) West Irish Sea, (E-IS) East Irish Sea and (SE-IS) Southeast Irish Sea. Contains OS data Crown copyright and database right 2018 and the GEBCO_2014 Grid, version 20150318, www.gebco.net.

From overall CPUE, the CPUE for age-0, age-1 and mature whiting was calculated per haul as the sum of the proportions of each 1 cm length class of that life stage. Proportions were modelled as the probability of an individual belonging to a given life stage using binomial generalised linear models (GLMs) with logit link. Sex-maturity-age-length keys (SMALKs) available as part of the DATRAS data set were used to fit the models. Individual models were fitted for each life stage for both the NIGFS (Eqs 2, 4 & 6) and SWC-IBTS (Eqs 1,3 & 5):

$$\begin{array}{l} logit(p0SWC_{i})=\alpha +\beta_{1}sex_{i}+\beta_{2}region_{i}+\beta_{3}length_{i}+\beta_{4}year_{i}+\beta_{5}(length_{i}\times region_{i})+\\ \beta_{6}(length_{i}\times year_{i})+\beta_{7}(sex_{i}\times year_{i}) \end{array}$$ (1)

$$\begin{array}{l} logit(p0IS_{i})=\alpha +\beta_{1}sex_{i}+\beta_{2}region_{i}+\beta_{3}length_{i}+\beta_{4}year_{i}+\beta_{5}(length_{i}\times region_{i})+\\ \beta_{6}(length_{i}\times year_{i})+\beta_{7}(sex_{i}\times region_{i})+\beta_{8}(region_{i}\times year_{i}) \end{array}$$ (2)

$$\begin{array}{l} logit(p1SWC_{i})=\alpha +\beta_{1}sex_{i}+\beta_{2}region_{i}+\beta_{3}length_{i}+\beta_{4}year_{i}+\beta_{5}(length_{i}\times region_{i})+\\ \beta_{6}(length_{i}\times year_{i})+\beta_{7}(region_{i}\times year_{i}) \end{array}$$ (3)

$$\begin{array}{l} logit(p1IS_{i})=\alpha +\beta_{1}sex_{i}+\beta_{2}region_{i}+\beta_{3}length_{i}+\beta_{4}year_{i}+\beta_{5}(length_{i}\times year_{i})+\\ \beta_{6}(region_{i}\times year_{i})+\beta_{7}(sex_{i}\times year_{i}) \end{array}$$ (4)

$$\begin{array}{l} logit(pmSWC_{i})=\alpha +\beta_{1}sex_{i}+\beta_{2}region_{i}+\beta_{3}length_{i}+\beta_{4}year_{i}+\beta_{5}(length_{i}\times sex_{i})+\\ \beta_{6}(region_{i}\times year_{i})+\beta_{7}(region_{i}\times sex_{i}) \end{array}$$ (5)

$$\begin{array}{l} logit(pmIS_{i})=\alpha +\beta_{1}sex_{i}+\beta_{2}region_{i}+\beta_{3}length_{i}+\beta_{4}year_{i}+\beta_{5}(length_{i}\times year_{i})+\\ \beta_{6}(region_{i}\times year_{i})+\beta_{7}(sex_{ij}\times year_{i})+\beta_{8}(sex_{i}\times length_{i}) \end{array}$$ (6)

where the probability *p*_0_, *p*_1_ and *p*_m_ of an individual i belonging to the given life stage is a function of length, sex, year, region and associated, significant interactions. To account for spatial variation in maturation schedules and length-at-age the variable categorising region was included. Regions displayed in Fig 1 were selected to reflect potential local scale differences in length-at-age and maturation schedules based on previous research [44,49–52]. Backwards stepwise selection using log likelihood ratio tests established the final optimal model structures. The midpoint of the probability curve at *p*_*0*,_ *p*_*1*_ and *p*_*m*_ = 0.5, from here on referred to as L_50_, is equivalent to 50% of the length class belonging to a given life stage. The L_50_ measures were used to identify spatial variation in length-at-age and maturation-at-length.

### Environmental variables

**Fig 2:**
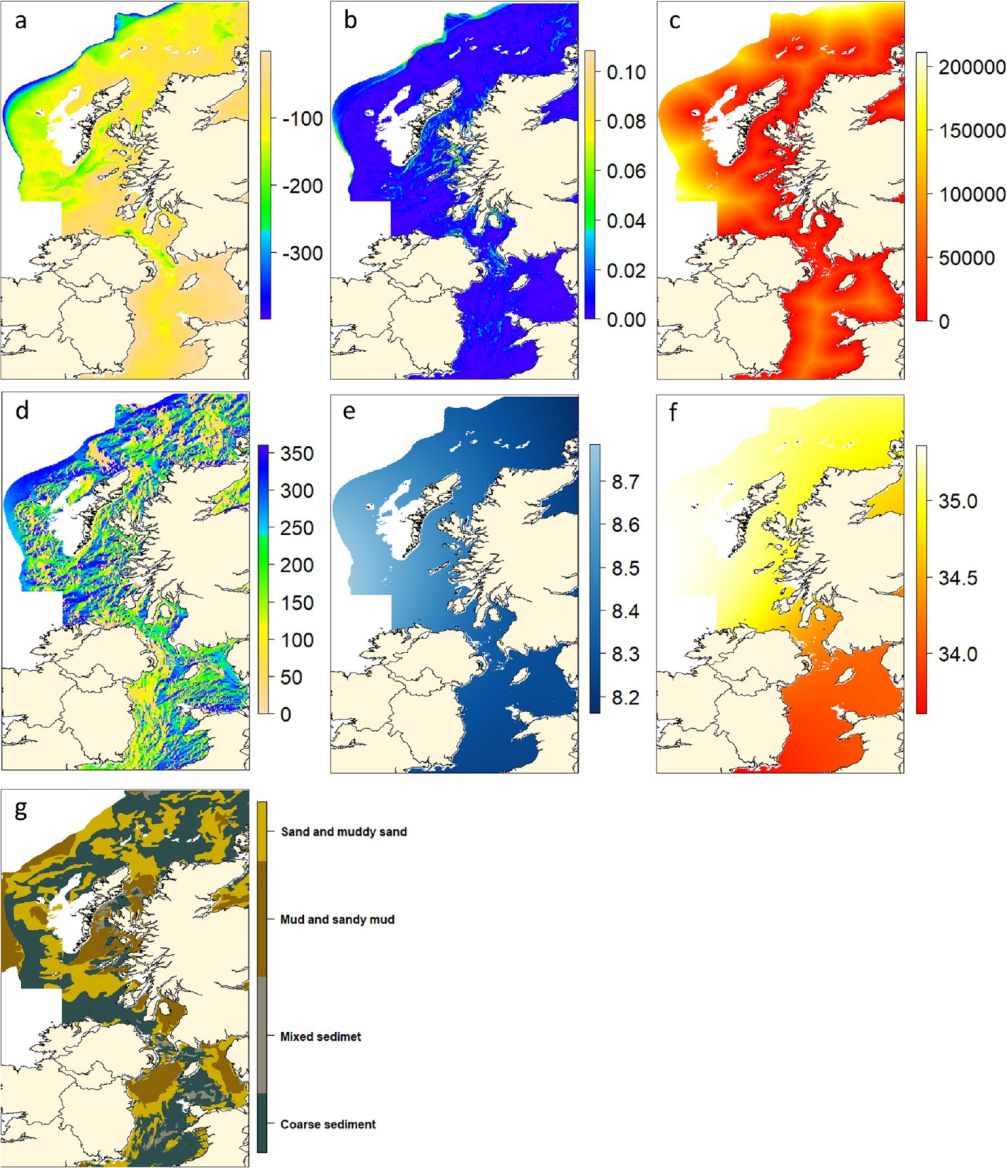
Summary of the explanatory environmental layers used in GAMM models of whiting life stage abundance. Extent of all layers dictated by the limits of available sediment data (longitude = 3°W to 10°W, latitude = 52°N to 59°N). (a) Bathymetry (m) to depths of 400m. (b) Slope gradient (rad). (c) Proximity to shore (m). (d) Slope aspect (° to N) (e) Mean bottom temperature Feb-March 2009 (°C). (f) Mean bottom salinity Feb-March 2009. (g) Sediment type. Contains OS data Crown copyright and database right 2018 and the GEBCO_2014 Grid, version 20150318, www.gebco.net.

Environmental data were collated in R 3.3.0 [53] using the *sp*, *raster* and *rgdal* packages [54–56]. The environmental layers included the temporally stable variables: bathymetry, slope gradient, slope aspect, proximity to shore and sediment type. All raster layers and matrices were constructed at a resolution of 928 x 1660 cells of area 1.54 km^2^. Fig 2 displays the environmental layers used in the analysis with examples of temperature and salinity from Feb-March 2009. The temperature and salinity layers from the other study time-points are presented in the supporting information S1 Fig. Bathymetry data was derived from the General Bathymetric Chart of the Oceans (GEBCO) and sourced from the British Oceanographic Data Centre [57]. The bathymetric data allowed the creation of associated layers for slope gradient and aspect (direction (0 to 360) faced by slopes with North being 0). Distance from the centre of cells to the nearest coastline was calculated using the *rgeos* package [58]. Data documenting substratum type was sourced from the British Geological Survey [59]. Categories in this substratum classification system were merged to bring the existing data in line with the EUNIS habitat classification system [60]. This reduced the original 17 categories to 5. Of these, “Rock” was not included in the analysis as this bottom type is inaccessible to the GOV gear used in the Scottish surveys. Temporally variable layers were constructed for relevant time points and included near bottom temperature and salinity. Near bottom temperature and salinity were extracted from conductivity, temperature, and depth (CTD) data available from the ICES Oceanography data portal [61]. The highest pressure and thus the closest to bottom measurements for a given station were used in the analysis. To ensure adequate spatial coverage, mean temperature and salinity data were calculated over the duration of the trawl surveys. Kernel smoothing, implemented from the *fields* package [62] was applied to the salinity and temperature data. An optimal bandwidth (θ) for the Gaussian kernel was selected by least squares, leave-one-out cross validation.

### Spatial data analysis

Distribution models were constructed using generalised additive mixed effects models (GAMM) to predict the abundance of age-0, age-1 and mature whiting across the UK west coast. Generalised additive models (GAM) have been widely used to model this type of data to account for nonlinear responses and better reflect ecological relationships [6,18,63–65]. A mixed effects modelling approach was adopted to account for different survey methods used across the study area. A two-part (zero-altered Poisson model (ZAP)) [66] approach was adopted to properly account for zero inflated data [67]. A binomial GAMM was used to model the probability of whiting presence-absence and a zero truncated Poisson GAMM fitted to the abundance data. Both components were modelled as functions of the environmental variables using mixed effects GAMs from the *gamm4* package [68]. Model selection for both binomial and Poisson components was conducted with backwards/forwards stepwise comparisons using AIC. Variance inflation factors (VIFs) were used to identify collinearity in the explanatory variables. VIF values >3 were removed from the model [66]. Eqs 7–12 show the optimal GAMMs for each life stage where *p* is the probability of presence and *n* is predicted abundance for haul *j* from survey *i* fitted as the random intercept *a*. One-dimensional smoothers (s) and two-dimensional tensor products (te), other than aspect, were fitted with penalised cubic regression splines. Aspect was fitted using a penalised, cyclic cubic regression spline.

$$\begin{array}{l} logit(pAge0_{ij})=\alpha +s(longitude{)}_{ij}+te(slope\times longitude{)}_{ij}\\ +te(proximity\;to\;shore\times longitude{)}_{ij}+a_{i}+\varepsilon_{ij} \end{array}$$ (7)

$$\begin{array}{l} nAge0_{ij}=\alpha +s(proximity\;to\;shore{)}_{ij}+s(temperature{)}_{ij}+\\ te(depth\times proximity\;to\;shore{)}_{ij}+te(depth\times longitude{)}_{ij}+\\ te(slope\times longitude{)}_{ij}+te(proximity\;to\;shore\times temperature{)}_{ij}+\\ se\dim ent+a_{i}+\varepsilon_{ij} \end{array}$$ (8)

$$\begin{array}{l} logit(pAge1_{ij})=\alpha +s(proximity\;to\;shore{)}_{ij}+te(depth\times longitude{)}_{ij}+\\ te(proximity\;to\;shore\times slope{)}_{ij}+te(temperature\times slope{)}_{ij}+te(longitude\times slope{)}_{ij}+\\ Year_{ij}+se\dim ent_{ij}+a_{i}+\varepsilon_{ij} \end{array}$$ (9)

$$\begin{array}{l} nAge1_{ij}=\alpha +s(depth{)}_{ij}+s(slope{)}_{ij}+s(aspect{)}_{ij}+s(temperature{)}_{ij}+\\ s(proximity\;to\;shore{)}_{ij}+te(depth\times slope{)}_{ij}+te(depth\times proximity\;to\;shore{)}_{ij}+\\ te(depth\times temperature{)}_{ij}+se\dim ent+Year+a_{i}+\varepsilon_{ij} \end{array}$$ (10)

$$\begin{array}{l} logit(pMature_{ij})=\alpha +s(depth{)}_{ij}+te(temperature\times slope{)}_{ij}+te(longitude\times slope{)}_{ij}+\\ te(depth\times temperature{)}_{ij}+te(proximity\;to\;shore\times slope{)}_{ij}+\\ te(proximity\;to\;shore\times aspect{)}_{ij}+te(proximity\;to\;shore\times longitude{)}_{ij}+\\ Year_{ij}+a_{i}+\varepsilon_{ij} \end{array}$$ (11)

$$\begin{array}{l} nMature_{ij}=\alpha +s(depth{)}_{ij}+s(temperature{)}_{ij}+s(longitude{)}_{ij}+\\ te(depth\times longitude{)}_{ij}+te(temperature\times proximity\;to\;shore{)}_{ij}+\\ te(slope\times temperature{)}_{ij}+Year+a_{i}+\varepsilon_{ij} \end{array}$$ (12)

Binomial, presence-absence model performance was assessed by calculating the area under the curve (AUC) for the receiver operator characteristic (ROC) curve using the *ROCR* package [69]. Abundance models, both Poisson and combined (Binomial component × Poisson component), were assessed for predictive performance using Spearman’s Rank Correlation Coefficient. All cross-validations were run with 100 iterations of a randomised 7:3 data split into training and tests sets respectively stratified by haul. The best performing models were used to construct relative abundance maps for each life stage and year.

To identify areas of high density and thus importance to specific life stages in each year, aggregation curves were constructed. Geostatistical aggregation curves relate the proportional abundance of fish to the area occupied by those fish. By ranking hauls by density from maximum to minimum the cumulative abundance as a proportion of the total can be calculated along with the proportion of the total area covered by the hauls. The curves describe the accumulation of abundance as area occupied increases. Furthermore, the aggregation curve can be used to identify the hauls (or cells in the predicted surface) with the highest rates of accumulation per unit area. Therefore, aggregations of individuals can be defined by a value derived from the abundance-area relationship rather than from an arbitrary population density. This allowed each grid cell in the study to be categorised as aggregated or dispersed following the methods of Petitgas [28] and Colloca *et al*. [70].

As described by Petitgas [28] and Colloca *et al*. [70] the tangent to the curve with a slope of 1 was used as a threshold to categorise space use as either aggregated or dispersed (i.e. not aggregated) (S2 Fig). Any tangent to the curve with a slope ≥1 corresponded to areas where an increase in abundance resulted in a minimal increase of occupied area and was considered aggregated. Tangents with slopes <1 indicated dispersed distributions, as small increases in abundance resulted in large expansions of occupied area. The percentile from the modelled abundance frequency distribution corresponding to the threshold value thus delineated cells containing densities above and below this threshold. Grid cells in the distribution maps were then identified as areas of aggregation and assigned a cell value of 1 if the modelled density was above the threshold. Cells containing values below the threshold were classified as dispersed and assigned a value of 0. The persistence of areas of aggregation was quantified by summing these values for each cell across all the years studied. Here we consider persistence to be a measure of the recurrence of aggregations in the same locations during the study period.

## Results

Length-at-age and length-at-maturity varied throughout the duration of the study period and between many of the 9 pre-defined areas (displayed in Fig 1 and S3 Fig). Accounting for the spatial and temporal variation in length-at-age and -maturity minimised over- or under-estimates of the abundance of each life stage in the scientific trawl survey samples. Significant differences were observed in the L_50_ in both sexes between the pre-defined regions (S1 Table). The greatest absolute difference between regions was 82 mm occurring between N Minch and E Irish Sea in age-0 females in 2011. Age-1 (four months later) and mature whiting showed similar average differences between regions. The maximum difference between regions observed in any study year was 101 mm in age-1 and 114 mm in mature females. The most consistent differences occurred between regions separated by substantial distances like the SE Irish Sea and the N and S Minch. However, differences also occurred between adjacent regions, for example, the S Minch and S West.

Cross-validation of model performance indicated that the presence-absence components of the age-1 and mature models had achieved AUC values >0.7. The age-0 model achieved an AUC of 0.54, only marginally above that achieved at random and below the threshold considered fail (0.6). Performance of the Poisson and combined model components assessed with Spearman’s correlation coefficient ranged from moderate (≥0.4) to strong (>0.6) (Tables 1–3).

**Table 1:** Variable importance and CV metrics of the GAMM distribution model predicting probability of presence and abundance of age-0 whiting.

Model/ variable	Binomial				Poisson			
	Δ deviance	edf/df	Chi-square	p-value	Δ deviance	edf/df	Chi-square	p-value
Sediment type	-	-	-	-	4.34	3	8.76	**0.032**
Temperature	-	-	-	-	10.51	2.48	18.69	**<0.001**
Longitude	2.79	2.72	21.51	**<0.001**	-	-	-	-
Proximity to shore	-	-	-	-	3.15	1.00	6.09	0.064
Proximity to shore: Temperature	-	-	-	-	2.50	1.78	5.00	0.097
Longitude: slope	1.36	3.78	8.37	0.138	4.81	1.71	8.81	**0.014**
Proximity to shore: longitude	2.47	12.39	19.97	0.124	-	-	-	-
Proximity to shore: depth	-	-	-	-	2.10	2.88	3.58	0.059
Depth: longitude	-	-	-	-	7.31	1.00	13.00	**<0.001**
Gear type (random)	0.43	0.92	3.25	0.050	20.29	0.97	47.10	**<0.001**
Model performance			AUC = 0.54		Spearman coefficient = 0.39 (p<0.001)			
Combined model performance					Spearman coefficient = 0.40 (p<0.001)			

Δ deviance given for variables in the binomial and Poisson model components showing the relative importance of each to the model. Cross-validation conducted on 100 iterations of random 3:7 (test: training) data splits for binomial and Poisson components and combined models. Significant p-values at p < 0.05 are in bold.

**Table 2:** Variable importance and CV metrics of the GAMM distribution model predicting probability of presence and abundance of age-1 whiting.

Model/ variable	Binomial				Poisson			
	Δ deviance	edf/df	Chi-square	p-value	Δ deviance	edf/df	Chi-square	p-value
Depth	-	-	-	-	2.36	1.53	7.54	**0.005**
Slope	-	-	-	-	6.47e^-07^	2.80e^-06^	0.000	0.420
Aspect	-	-	-	-	3.76	2.91	15.11	**0.001**
Proximity to shore	7.63	1.00	30.66	**<0.001**	5.74	2.67	26.18	**<0.001**
Temperature	-	-	-	-	2.45	3.49	10.48	**0.014**
Sediment type	4.88	3.00	2.70	**0.007**	1.83	3.00	9.71	**0.021**
Year	8.82	6.00	30.87	**<0.001**	4.12	6.00	17.99	**0.006**
Depth: slope	-	-	-	-	2.42	3.83	8.20	**0.044**
Depth: longitude	13.30	10.76	42.16	**<0.001**	-	-	-	-
Depth: temperature	-	-	-	-	1.64	1.68	5.21	**0.024**
Slope: temperature	6.49	4.00	25.76	**<0.001**	-	-	-	-
Slope: longitude	0.49	1.84	2.08	0.581	-	-	-	-
Proximity to shore: slope	6.92	4.55	20.29	**0.002**	-	-	-	-
Depth: Proximity to shore	-	-	-	-	0.61	1.22	1.66	0.207
Gear type (random)	6.20	1.00	16.11	**<0.001**	1.47	0.78	6.86	**0.002**
Model performance		AUC = 0.88			Spearman coefficient = 0.50 (p<0.001)			
Combined DG model performance		-			Spearman coefficient = 0.57 (p<0.001)			

Δ deviance given for variables in the binomial and Poisson model components showing the relative importance of each to the model. Cross-validation conducted on 100 iterations of random 3:7 (test: training) data splits for binomial and Poisson components and combined models. Significant p-values at p < 0.05 are in bold.

**Table 3:** Variable importance and CV metrics of the GAMM distribution model predicting probability of presence and abundance of mature whiting.

Model/ variable	Binomial				Poisson			
	Δ deviance	edf/df	Chi-square	p-value	Δ deviance	edf/df	Chi-square	p-value
Depth	4.55	1.00	12.866	**<0.001**	1.88	1.92	5.15	0.093
Longitude	-	-	-	-	1.07	1.00	3.91	**0.048**
Temperature	-	-	-	-	3.37	1.00	13.53	**<0.001**
Year	20.27	1.97	2.885	**0.004**	9.74	6.00	51.90	**<0.001**
Depth: longitude	-	-	-	-	4.25	3.78	15.59	**0.009**
Depth: temperature	2.53	1.99	4.863	0.111	-	-	-	-
Slope: proximity to shore	3.28	1.00	4.538	**0.033**	-	-	-	-
Slope: temperature	0.05	1.01	0.044	0.842	1.49	1.00	4.49	**0.034**
Slope: longitude	1.90	3.11	4.459	0.363	-	-	-	-
Temperature: proximity to shore	-	-	-	-	2.70	2.43	7.01	**0.044**
Proximity to shore: aspect	8.24	6.98	16.575	**0.011**	-	-	-	-
Proximity to shore: longitude	4.82	3.32	9.685	**0.037**	-	-	-	-
Gear type (random)	3.60e^-06^	5.93e^-06^	0.001	0.736	6.02	0.95	22.30	**<0.001**
Model performance		AUC = 0.78			Spearman coefficient = 0.44 (p<0.001)			
Combined DG model performance		-			Spearman coefficient = 0.45 (p<0.001)			

Δ deviance given for variables in the binomial and Poisson model components showing the relative importance of each to the model. Cross-validation conducted on 100 iterations of random 3:7 (test: training) data splits for binomial and Poisson components and combined models. Significant p-values at p < 0.05 are in bold.

**Fig 3:**
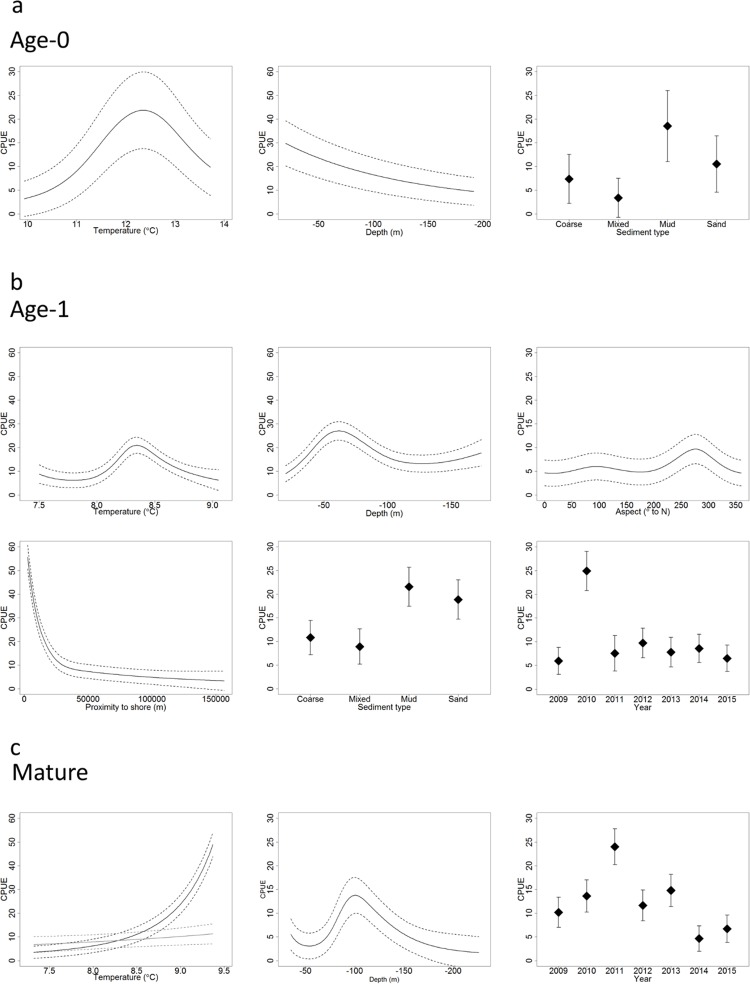
Combined GAMM predicted responses of influential fixed effects environmental variables in age-0, age-1 and mature whiting. Variable effect displayed on the scale of the response variable (Catch Per Unit Effort (CPUE)) (a) Variable effects on age-0 abundance at mid longitude and mean proximity to shore. Temperature and depth effects predicted on mud sediment type. Sediment type effect predicted at mean temperature and depth. (b) Variable effects on age-1 abundance at mid longitude and mean proximity to shore in 2014. Temperature depth and aspect effects predicted on mud sediment type. Sediment type and year effect predicted at mean temperature and depth and 270° aspect. (c) Variable effects on mature whiting abundance. Depth and year predictions at mid longitude, mean proximity to shore, slope and temperature and at 270° aspect in 2014. Two temperature predictions (black and grey lines) are shown depending on the effects of slope and proximity to shore. Black lines show the response to temperature for shallow slopes (~0 rad) far from shore (>34 km). Grey lines show the response to temperature for steep slopes (>0.001 rad) close to shore (<2.7 km).

The importance of each variable to the model can be seen in Tables 1–3 along with their associated summary output. The environmental variables most influencing local abundance in age-0 whiting were temperature and, depending on its spatial interaction with longitude, depth. Increasing depth had a negative effect on densities at mid longitudes. This effect reversed further west or east. Slope gradient also influenced age-0 densities dependent on longitude. Elevated densities occurred over the finer sediment types of “sand and muddy sand” and “mud and sandy mud”. The overall effect of temperature shows highest densities occurring from 12 to 13°C varying with distance from shore while densities also decrease with increasing depth (Fig 3A).

Proximity to shore was the most important explanatory variable in the age-1 model followed by “year”. Locations close to shore (within 30 km) exhibited the strongest positive influence on age-1 whiting numbers. Other influential variables included: aspect, depth, temperature and sediment type. The probability of presence of age-1 whiting was strongly influenced by depth given longitude and their proximity to shore. Similar to the age-0 model, finer sediment types resulted in higher predicted abundances of age-1 whiting. Optimal depth varied with slope, proximity to shore and temperature with the highest abundances occurring at 60 m. Lower values of slope and proximity to shore resulted in an increased effect of depth on predicted age-1 whiting abundance while lower temperatures resulted in higher abundances occurring in shallower water (50 m). Given the cooler overall temperatures during these surveys from February to March and, allowing for the interaction with depth, temperatures of 8 to 8.5°C appeared optimal (Fig 3B).

In the mature whiting model, “year” was among the most important variables, but of the environmental variables, depth and temperature were most influential. Interactions were present between depth and longitude with positive effects of depth on abundance observed at 50–60 m in more easterly areas while further west and offshore the positive depth effect was seen at 100 m. Higher abundance was associated with higher temperatures up to 9.7°C, higher than that seen for the same time of year in the age-1 whiting model. Interactions were present between temperature and proximity to shore, and temperature and slope gradient. The increase in abundance resulting from higher temperatures was maximised on shallow slopes far from shore (Fig 3C).

The random effect of fishing gear type was retained during model selection in both binomial and Poisson components in all three life stage distribution GAMMs. In each of the three binomial model components gear type, fitted as a random effect, was the least influential variable or among the lowest (3^rd^ least influential of 8 variables for the age-1 model). In the Poisson (abundance) component of the age-1 model the importance of this variable is also relatively low (3^rd^ lowest of 11 variables). This contrasts with the much more substantial effect seen in the Poisson component of the age-0 and mature whiting models. The importance of the random effect in the Poisson components for age-0 and mature whiting is among the highest (ranked 1^st^ and 2^nd^ respectively). In these cases, the high importance of the random effect is indicative of the high variability between the gear types also indicating that this effect is consistent. However, in models displaying a low influence from the random effect there may be little difference in the effect of gear type or the effect may not vary consistently across samples. It is important to note that the gear type used is likely to co-vary with several other variables (not included in the models). As described earlier in the methods rock-hopper otter trawls are used predominantly in the Irish Sea while in Scottish west coast surveys GOV trawl gear is used. This inevitably means that the effects of the boat used, crew and geographic region will also influence the random effect named “gear type.”

**Fig 4:**
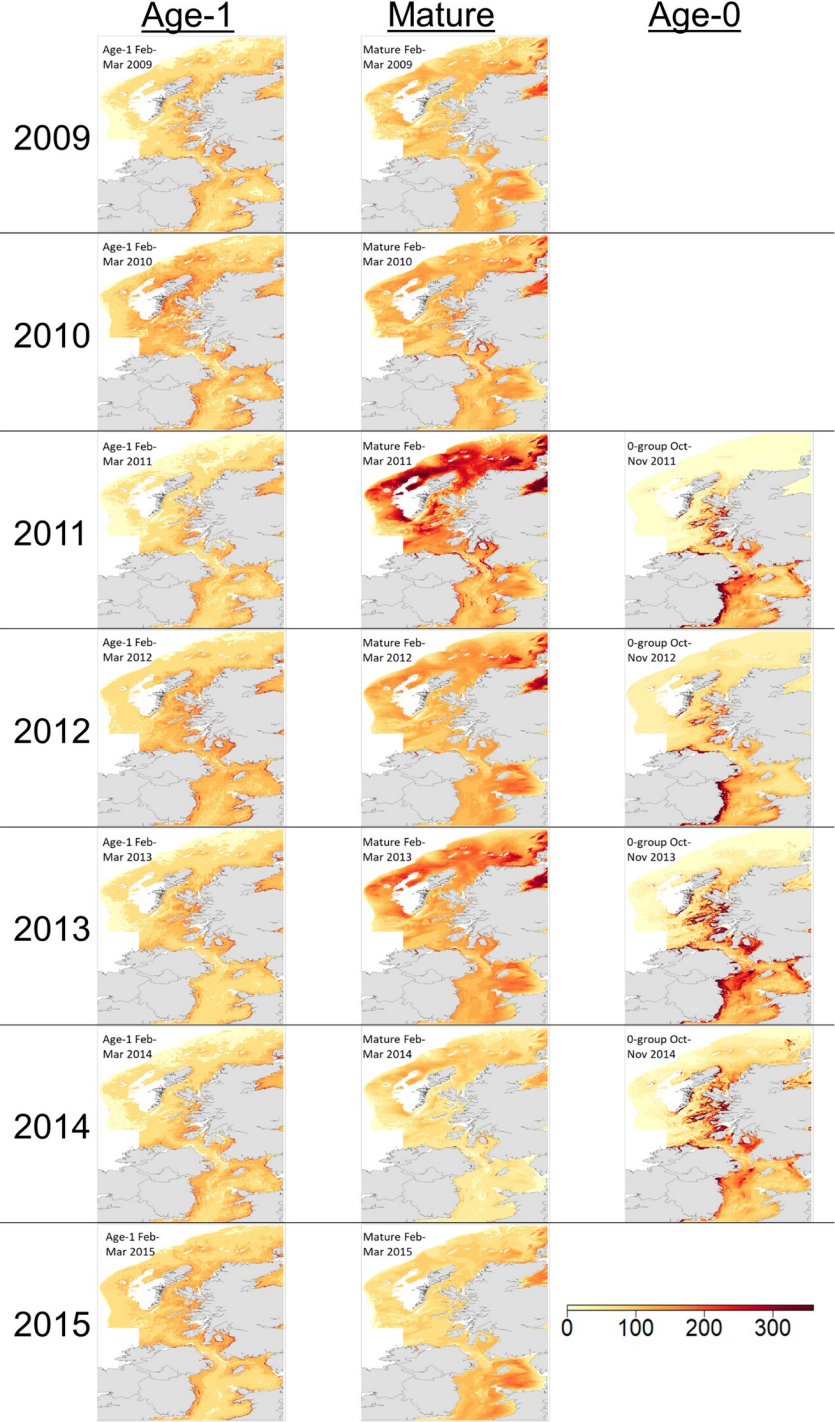
Mapped predicted relative abundance of whiting at key life stages. Relative abundance is Catch Per Unit Effort (CPUE) with the effect of gear type controlled for as a random effect. Modelled time points are early spawning season (February to March) for age-1 and mature fish and late recruitment for the age-0 (October to November). Contains OS data Crown copyright and database right 2018 and the GEBCO_2014 Grid, version 20150318, www.gebco.net.

Life stage specific modelled abundance is shown in Fig 4. The age-0 model clearly shows areas of highest density near shore along the west coast of Scotland and the eastern Irish coast. Four months later, age-1 abundance maps show fewer patches of very high density in comparison to those of age-0. Areas further from shore and in deeper water previously unoccupied by age-0 fish were occupied by age-1 whiting. The relative abundance of mature whiting showed much more year to year fluctuation. The ontogenetic shift towards occupying areas in deeper water, further from shore continued from that seen in the age-1 model.

**Fig 5:**
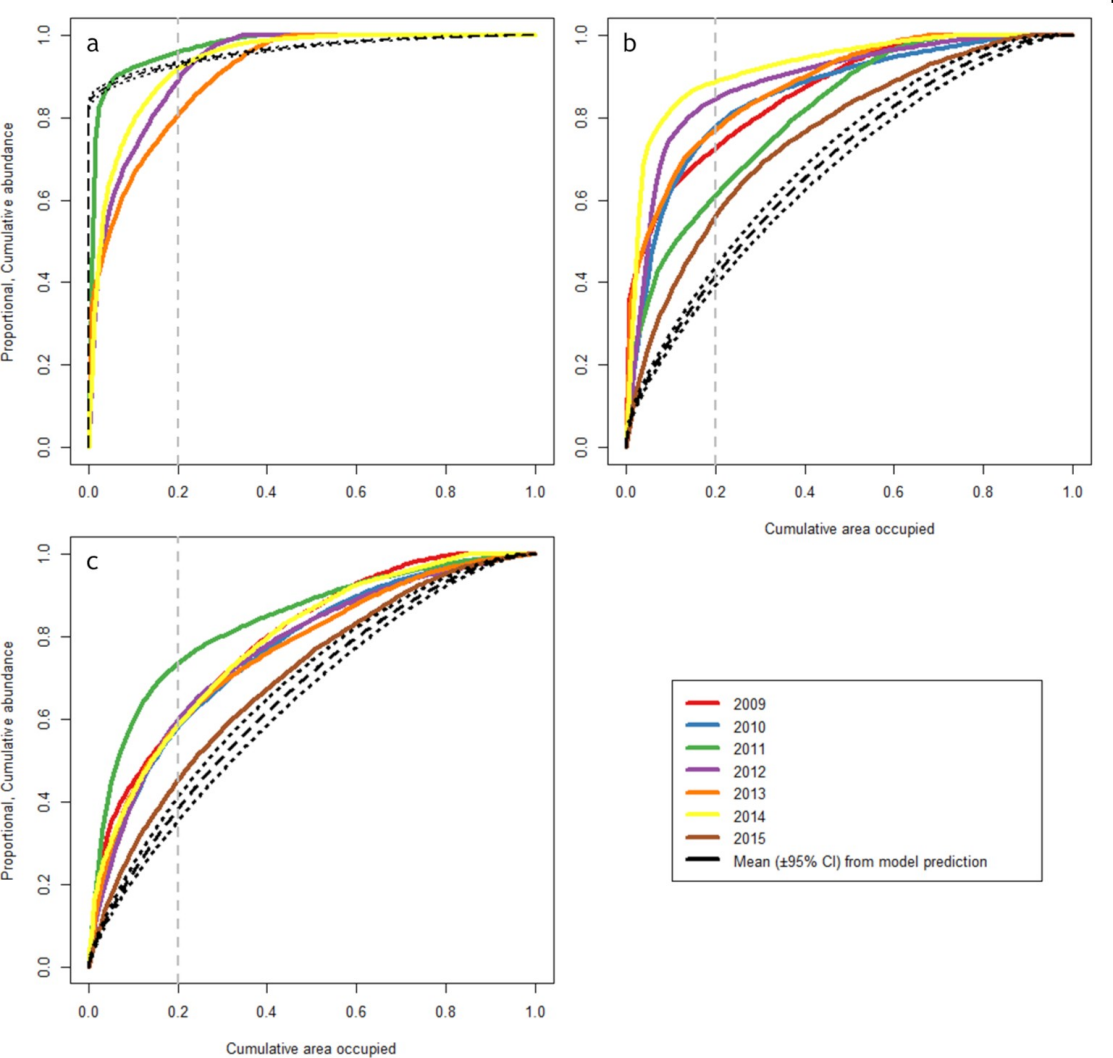
Geostatistical aggregation curves showing highly clustered age-0 whiting and more defuse later life stages. Lines show proportional cumulative abundance increasing as a function of the proportion of the occupied area for: (a) age-0, (b) age-1 and (c) mature whiting. Dashed black lines display the mean (± 95% CI) of the model prediction. Dashed, vertical grey lines display a 20% occupied area reference line.

The aggregation curves indicate between 80% to 96% of the age-0 fish are contained in only 20% of the space occupied (Fig 5A). The mean model prediction for age-0 whiting (Fig 5A grey lines) suggests 93% of the fish occupy 20% of the utilised area. Progressing through the life cycle, age-1 whiting (Fig 5B) conform to a more dispersed pattern with 56% to 88% of the annual fish abundance spread throughout 20% of occupied space. This more dispersed pattern of occupation continues in mature whiting (Fig 5C) where 45% to 73% of fish are dispersed over 20% of the space occupied. The model predictions for the age-1 and mature whiting life stages underestimate the space selectivity (Fig 5B and 5C). The age-1 model predicted 42% of this age group to be contained in 20% of the occupied area and the mature model predicted 38% to be dispersed throughout 20% of the area occupied.

**Fig 6:**
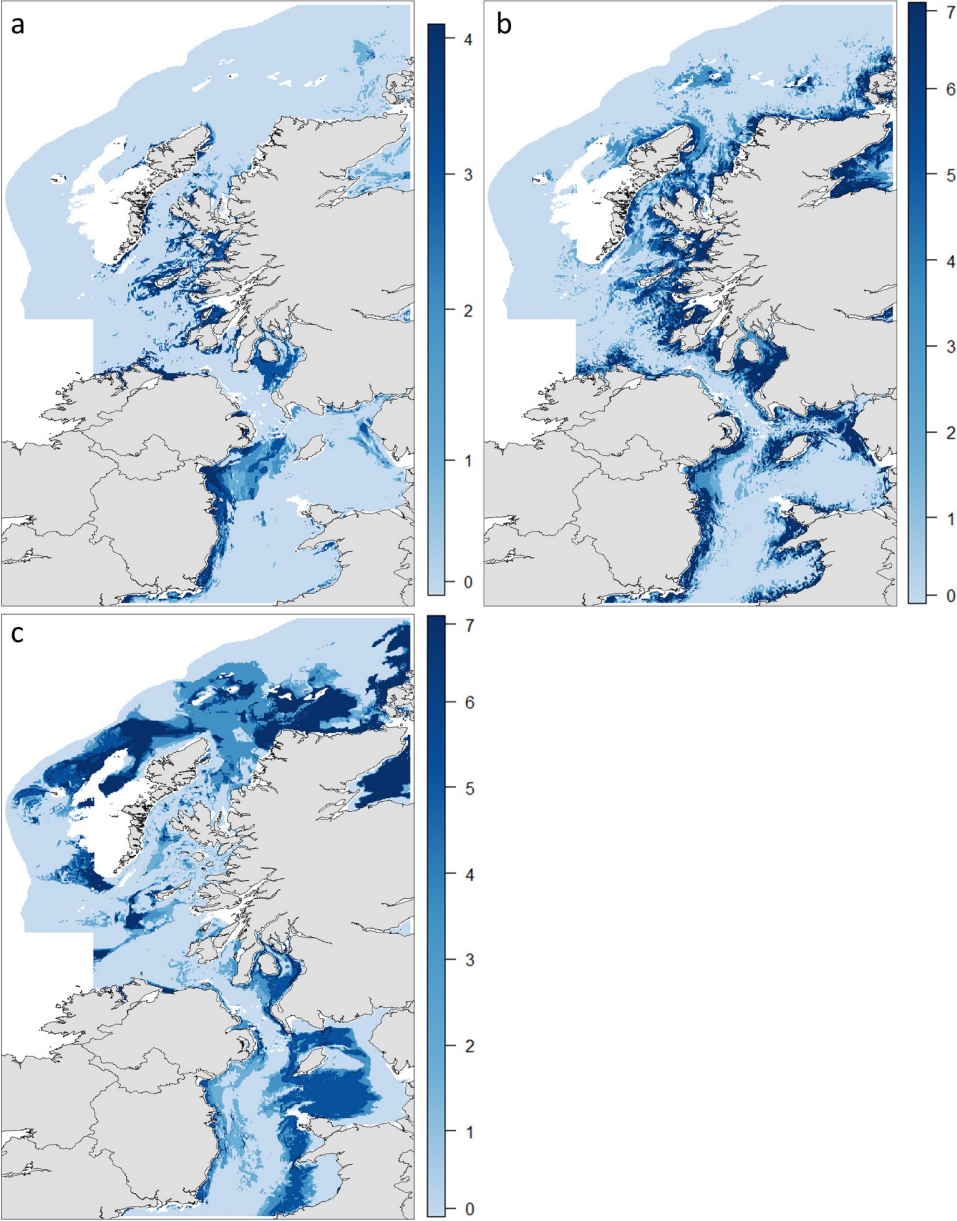
Persistent areas of whiting aggregation at three life stages. Scale bars indicate the number of years in which given areas were classified as containing aggregations of whiting. (a) age-0 whiting in October and November 2011–2014. (b) Age-1 whiting in February and March 2009–2015. (c) Mature whiting in February and March 2009–2015. Note: panel (a) shows 0–4 years, while panel (b) and (c) display from 0–7 years. Contains OS data Crown copyright and database right 2018 and the GEBCO_2014 Grid, version 20150318, www.gebco.net.

The classification of space use allowed the aggregations at each life stage to be mapped (S4 Fig). Table 4 displays a measure of precision for this classification. Precision in this case is measured as the percentage of the total number of cells falling out with a 5% error band around the classification threshold under the density distribution curve. Distinct differences in the geographic areas are again visible throughout the life cycle. By combining these data at each life stage, the persistence of areas of aggregation during the study was described and is displayed in Fig 6. Areas of aggregation were more temporally persistent for age-0 and age-1 than mature whiting. Several areas, particularly close to the fjordic sea lochs of the Scottish west coast and across the eastern Irish coast show consistently recurring aggregations of age-0 whiting. The areas of importance to age-1 whiting, not only include the previously occupied age-0 locations but also extend further offshore and along the coast. Several of these same coastal areas show persistent aggregations of age-1 whiting at the start of the spawning season. Additionally, the central and eastern Irish Sea gains greater importance while areas to the northwest of Scotland are less frequently utilised. Mature aggregations, although more dispersed by comparison to earlier life stages, recur most often offshore to the Scottish northwest and north of Ireland. Some coastal areas including the Firth of Clyde periodically show mature aggregations present. The mature whiting life stage includes a proportion of mature age-1 fish and thus some overlap exists between the aggregations of age-1 and mature fish. Within the Irish Sea the central basin is the most consistent area of aggregation. However, this does only account for a persistence during 50% of the study period.

**Table 4:** High relative sensitivity for the classification of whiting aggregations at age-0, age-1 and mature life stages.

Age-0 model	% of cells out with ±0.025 of threshold value	Age-1 model	% of cells out with ±0.025 of threshold value	Mature model	% of cells out with ±0.025 of threshold value
-	-	2009	98.26	2009	98.12
-	-	2010	98.27	2010	98.22
2011	>99.99	2011	98.22	2011	98.38
2012	>99.99	2012	98.27	2012	98.22
2013	99.98	2013	98.27	2013	98.25
2014	99.64	2014	98.29	2014	98.38
-	-	2015	98.25	2015	98.13

Sensitivity is displayed as the percentage of the total surface area contained in the 95% confidence window. The window is defined as areas under the density distribution out with a 0.05 error band with 0.025 above and 0.025 below the classification threshold for the age-0, age-1 and mature whiting models.

## Discussion

Substantial ontogenetic distribution shifts were observed in whiting in this study. From settlement to spawning, these changes result in a net movement from coastal to offshore waters. During our study persistent aggregations of age-0 whiting occurred in coastal areas to the west of the UK. The age-0 aggregations then dispersed and the extent of the area occupied by each cohort of whiting increased. As fish matured, the coastal Scottish areas became less frequently occupied in favour of areas further offshore. A similar, although less pronounced pattern can be seen in the Irish Sea.

The importance of offshore areas to mature whiting in this study is consistent with historically reported landings of larger fish being caught in these offshore areas [71]. Similar patterns of ontogenetic shifts in distribution have been reported in the North Sea. For example, Loots *et al*. [72] report a segregation of age classes with young fish occupying shallower water while older fish were distributed in deeper areas. On the UK west coast, we found westerly facing slopes predicted higher local abundances of age-1 whiting. Slopes orientated in this direction not only face the prevailing currents and tidal streams but also transition to deeper waters to the west. This may be indicative of age-1 whiting dispersing offshore to occupy deeper water.

The findings we report differ substantially from the maps of whiting nursery and spawning grounds presently used to advise marine developments [73]. The offshore areas identified in our study as consistently occupied by mature whiting at high densities are absent from those maps currently used. Given the high densities of large, mature fish in these offshore waters at the start of the spawning season, these areas are likely to be important for spawning whiting. The present work also differs from the currently used maps displaying a more widespread distribution of juveniles at high densities across the inshore Scottish west coast. We found some inshore areas, like the Firth of Clyde, harboured localised aggregations of mature fish in addition to other life stages. The presence of mature fish may result from a proportion of older fish remaining in the area rather than moving offshore or may be due to earlier maturation among whiting in this area [52]. The methodology we used to calculate the life stage proportions accounted for spatial variability in maturation schedules and was capable of modelling the presence of early maturing fish in this region. Hunter *et al*. [52] report particularly high rates of decrease in the maturation schedules of age-1 whiting in the Firth of Clyde and attribute this to selective pressure from size-selective trawl fishing.

The progression of whiting to waters further offshore at later life stages was accompanied by a reduction in dependence on finer sediments. This may be indicative of ontogenetic shifts in foraging strategy and preference for specific prey species. Dietary changes have been described throughout the life cycle of whiting, progressing from a combination of invertebrates and small fish to diets which are predominantly piscivorous [74–76]. The prey species taken by age-0 whiting are in many cases more closely associated with finer sediments. For example, 89% of the fish species found in the stomachs of small (100–149 mm) whiting were sandeels, blennies or gobies [75,77,78].

The response to temperature described in this paper also varied at each life stage. In age-1 west coast whiting, elevated abundances were observed at higher temperatures (8.3°C) than previously reported for North Sea whiting [79]. In mature whiting two modes of effect in response to temperature were observed. Far from shore, warmer temperatures resulted in increased densities. However, closer to shore, temperature had no effect on local abundance. Although inshore temperature regimes are likely to be more dynamic than those offshore, this type of response may result from two discrete modes of behaviour. This response observed in the model may be indicative of inshore foraging and spawning further offshore or targeting of different prey types [80,81].

Physical anthropogenic impacts on marine habitats notably include changes in temperature regimes through climatic change and the disruption of sea bed substrata from fishing activity [82–84]. González-Irusta and Wright [65,85] discuss the influences of these variables on spawning cod and haddock which both avoid fine, muddy sediments and seek out temperatures of 7°C and below. However, the disruptive effects of warming waters and fine, easily disturbed sediments may have less impact on whiting compared to other gadoid species. The differing responses are informative in the context of both climate change and increased particle suspension resulting from seabed disturbance [86,87].

We observed a positive effect from temperatures ≥ 7.5°C on mature whiting densities in offshore waters. Whiting are income breeders and feed during the spawning season [43]. In comparison to cod and haddock, whiting spawn later in the annual secondary production cycle to optimise the availability of food during spawning and for their offspring. Cod and haddock are capital breeders spawning early in the annual secondary production cycle when temperatures are 7°C or lower to maximise offspring prey availability. Whiting are naturally likely to experience warmer water temperatures during spawning. Therefore, disruptive effects of warming sea temperatures on spawning may be less critical for whiting than for cod or haddock. However, any direct influence of warming seas may also be less obvious because of the requirement for whiting to balance both spawning activity and feeding.

In our study, the distribution of mature whiting at the beginning of the spawning season was unaffected by sediment type. The avoidance of readily suspended sediments in cod and haddock is presumably the result of their specific courtship behaviours both of which involve lekking and extensive vocalisation [88]. Whiting not only lack the musculature to produce these calls but spawning events also occur higher in the water column than in other gadoid species [89]. This may go some way to explain the lack of influence of sediment type on mature whiting at the beginning of the spawning season.

The life stage specific models presented here perform well when compared to other studies using distribution models to predict gadoid abundance. Our reported Spearman’s rank correlation statistics are comparable or better than those seen in studies of cod egg distribution [64] as well as later life stage models of haddock and cod [6,65,85]. The present study did not directly account for biotic influences like competition which will likely influence distributions in years of particularly high abundance. However, as recognised by Shepherd and Litvak [24], spatial differences in measures such as length-at age are inconsistent with the concept of the ideal free distribution which is required to explain distributions using purely density-dependent mechanisms. Moreover, three previous studies modelling gadoid distributions (including specifically whiting but in the North Sea) found no evidence for spatial density dependent processes despite directly testing for its influence [6,85,90].

Use of less preferred habitat due to competition is likely to be minimal over the study period given the depleted stocks which are substantially below the ICES spawning stock biomass limit reference point (B_lim_) in both Scottish and Irish Sea areas [35,40]. Therefore, it is reasonable to presume that the environmental variables influencing life stage densities in whiting reported here are driving the observed distributions or are suitable proxies for unmeasured variables.

Fishing mortality has also been suggested as a potential driver of ontogenetic shifts in distribution. For example, Frank *et al*. [91] argue that the pattern for older, larger fish to progressively occupy deeper water may be a direct result of harvesting. By comparison to the age-0, age-1 and mature whiting modelled in our study, the age groups of cod considered by Frank *et al*. [91] represent predominantly mature fish. Any ontogenetic deepening responses are likely to be stronger in the earlier age groups which we consider. Recent work has analysed several Northeast Atlantic stocks, including whiting on the Scottish west coast, and found no evidence for ontogenetic shifts to deeper water being driven by fishing intensity [92]. Therefore, the ontogenetic changes in distribution to deeper water we report for whiting likely result from habitat preference rather than being an artefact of higher fishing mortality in shallower areas.

The areas of persistent aggregations we describe, indicate the importance of specific geographic locations to each of the three life stages. The areas identified as persistently occupied by age-0 whiting conform to a broad definition of nursery areas [70]; they persist for the duration of the life stage and in this case, only partially overlap with areas of adult occupancy. However, a nursery area is only important to the wider population if it successfully contributes recruits to adult populations [93]. Significant contributions of recruits to adult populations have been shown to arise from nurseries which are highly spatio-temporally stable [70]. The juvenile distributions described here in the Firth of Clyde, western Irish Sea and inshore Scottish west coast waters may therefore encompass the most important nursery areas. Previous work using otolith microchemistry has detected high levels of recruitment from northern Scottish west coast nurseries to adult areas north of the Hebrides [46]. Future research is required to quantify the extent of this connectivity from nurseries further south and to the adult areas described here further west off the Scottish coast.

The identification of important geographic areas across the life cycle of a species unlocks the possibility of spatio-temporal targeting or protection of specific life stages. Fisheries harvesting increases mortality and, depending on gear type, often targets large, older individuals [94–96]. Extraction of these individuals and selection for small, early maturing fish has resulted in Fisheries-Induced Evolution (FIE) occurring in several species [97,98]. FIE will likely have adverse impacts on stock yields in the long term, resulting in reduced mean sizes of fish caught, decreasing the stability and productivity of fisheries and could limit stock recovery [94,95,99–104].

To alleviate the widely-recognised effects of FIE and reverse trends for truncated population demographics, more informed fishing practices are required [94,95]. Mitigating the effects of FIE with spatial management measures requires sound knowledge of spatial occupancy patterns at all sizes and age-classes [95,105]. This understanding is of critical importance to implement informed, age-structured fishing. Models which accurately describe ontogenetic patterns of space use can inform legislation to alleviate pressure on particularly vulnerable or important life stages in specific geographic areas [104,106]. For example, a dome-shaped selectivity curve may be desirable to protect both small and large individuals focussing fishing effort on intermediate sized fish [95,107]. Using knowledge of life stage distributions, like that presented here, could enable more precise, age targeted fishing and limit extraction of small and large individuals by directing effort away from the areas where these fish aggregate.

Current European legislation requiring the landing of bycatch will undoubtedly result in several species becoming “choke species”. The term “choke species” has been coined to describe species for which a quota is reached, denying vessels the opportunity to catch other species [108]. This scenario is credible in the case of whiting off the west coast of the UK, which are primarily bycatch, caught in a profitable *Nephrops norvegicus* fishery. The life stage focussed modelling approach used here may therefore aid fisheries management and be employed to benefit fishers. Given the ontogenetic variation in whiting distributions, limiting fishing in coastal areas late in the calendar year would avoid the high-density early life stages and go some way to reduce whiting bycatch. Furthermore, the high spatial and temporal resolution made possible by the methods described here may negate the need for large areas to be closed permanently.

The techniques used in this paper could also be readily applied to other species where abundance and demographic data are available. Where vulnerabilities at specific life stages are identified, our approach could be used to address several challenges facing fisheries management. Particularly in species where ontogenetic changes in distribution are observed, the method described here could enable an age targeted management approach. By targeting specific age groups conservation goals can be achieved by protecting important life stages while allowing harvesting of more appropriate age classes. This knowledge can be used to reduce pressure at specific life stages, avoid problematic bycatch scenarios and counterbalance effects like fisheries induced evolution.

## Supporting information

S1 Fig Additional temporally variable environmental layers used in GAMM models of whiting life stage abundance. Extent of all layers dictated by the limits of available sediment data (longitude = 3°W to 10°W, latitude = 52°N to 59°N). (a) Mean bottom temperature Feb-March 2010 (°C). (b) Mean bottom salinity Feb-March 2010. (c) Mean bottom temperature Feb-March 2011 (°C). (d) Mean bottom salinity Feb-March 2011. (e) Mean bottom temperature Feb-March 2012 (°C). (f) Mean bottom salinity Feb-March 2012. (g) Mean bottom temperature Feb-March 2013 (°C). (h) Mean bottom salinity Feb-March 2013. (i) Mean bottom temperature Feb-March 2014 (°C). (j) Mean bottom salinity Feb-March 2014. (k) Mean bottom temperature Feb-March 2015 (°C). (l) Mean bottom salinity Feb-March 2015. (m) Mean bottom temperature Oct-Nov 2009 (°C). (n) Mean bottom salinity Oct-Nov 2009. (o) Mean bottom temperature Oct-Nov 2010 (°C). (p) Mean bottom salinity Oct-Nov 2010. (q) Mean bottom temperature Oct-Nov 2011 (°C). (r) Mean bottom salinity Oct-Nov 2012. (s) Mean bottom temperature Oct-Nov 2013 (°C). (t) Mean bottom salinity Oct-Nov 2013. (u) Mean bottom temperature Oct-Nov 2014 (°C). (v) Mean bottom salinity Oct-Nov 2014. (w) Mean bottom temperature Oct-Nov 2015 (°C). (x) Mean bottom salinity Oct-Nov 2015. Contains OS data Crown copyright and database right 2018 and the GEBCO_2014 Grid, version 20150318, www.gebco.net.
(PDF)

S2 Fig Method of classifying spatial occupancy patterns using aggregation curves. (a) Example aggregation curve plotting cumulative proportional abundance against the proportional area occupied. (b) Cubic polynomial fitted to the transition phase of the curve. (c) Derived tangent to the curve with a slope of 1 identifying the point at which space use changes from aggregated to dispersed. (d) x and y coordinates of the point where the tangent meets the curve. Y indicates the aggregated proportion of the fish distribution and thus the percentile “cut-off” to apply to the overall density distribution while x shows the proportion of total space occupied by these fish.
(TIF)

S3 Fig Geographic areas referred to in the text. Dotted lines indicate the boundaries defining regions used in the analyses. Contains OS data Crown copyright and database right 2018 and the GEBCO_2014 Grid, version 20150318, www.gebco.net.
(TIF)

S4 Fig Areas of whiting aggregation at 3 key life stages. Dark blue areas delineate aggregations as defined by the threshold value derived from geostatistical aggregation curves. Contains OS data Crown copyright and database right 2018 and the GEBCO_2014 Grid, version 20150318, www.gebco.net.
(TIF)

S1 Table Whiting length-at-age and length-at-maturity (L_50_, cm). Age-0 were modelled from October—November surveys and show the length at which the probability a fish being age-0 = 0.5. Below this length, the probability of a fish belonging to this group increases. Age-1 and mature models were constructed from surveys conducted between February and March. The probability of fish being age-1 increases for values below L_50_. Conversely, mature fish above L_50_ are more likely to be mature.
(PDF)
