# Supplementary figures and images for: A method to improve fishing selectivity through age targeted fishing using life stage distribution modelling
Source: PLoS One. 2019 Apr 2;14(4):e0214459. doi: 10.1371/journal.pone.0214459 (PMC6445474; doi:10.1371/journal.pone.0214459)

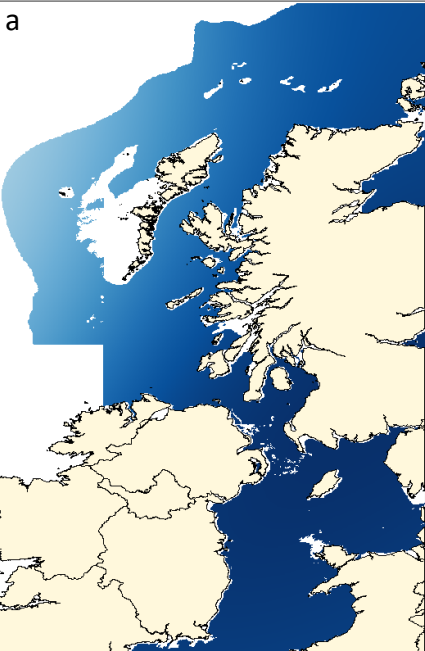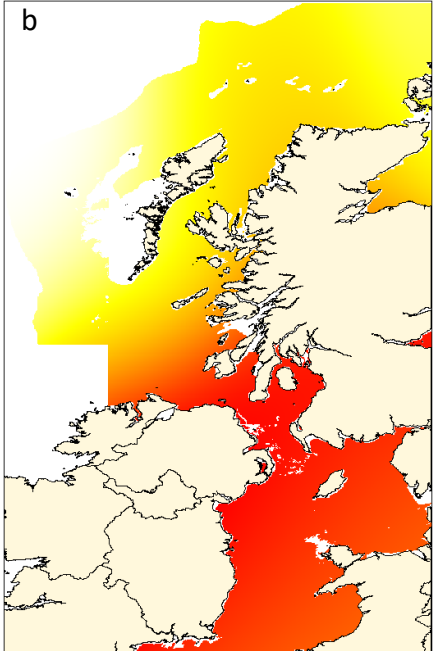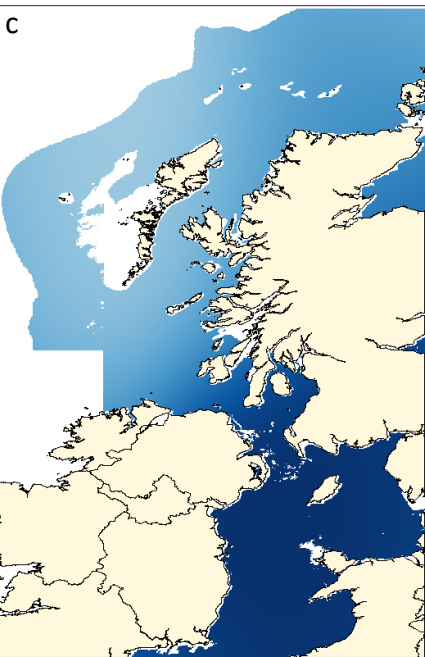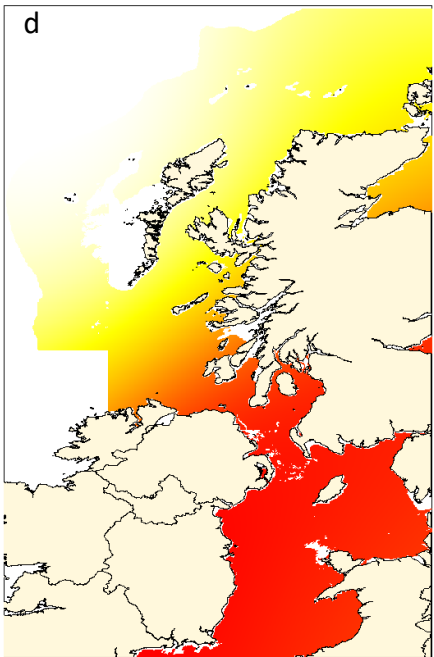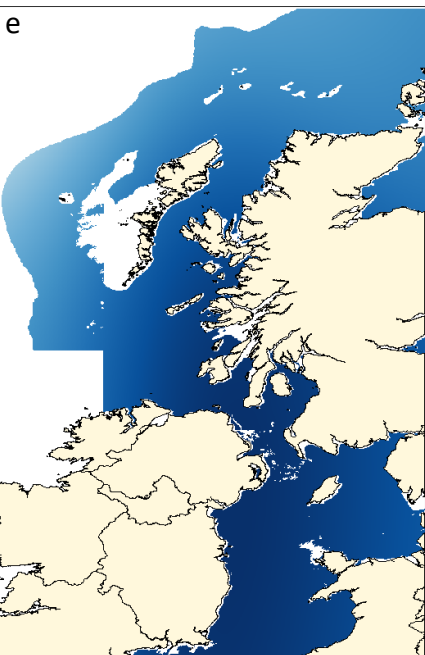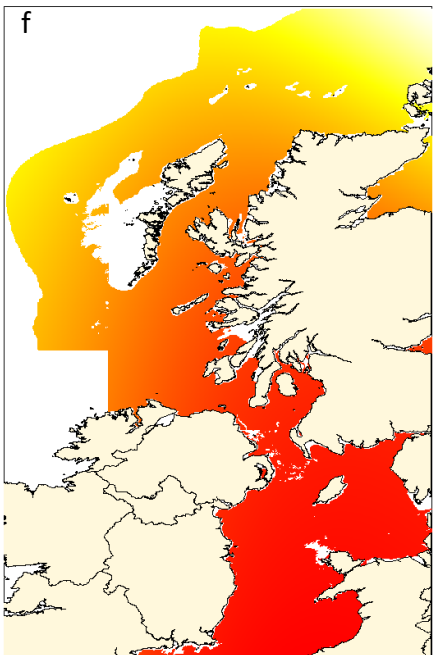

g

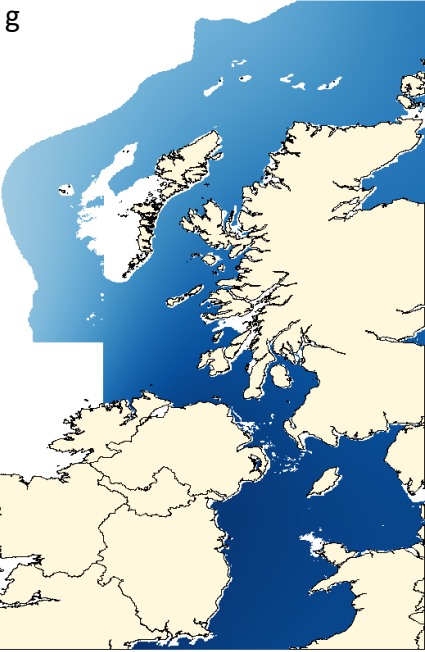

h

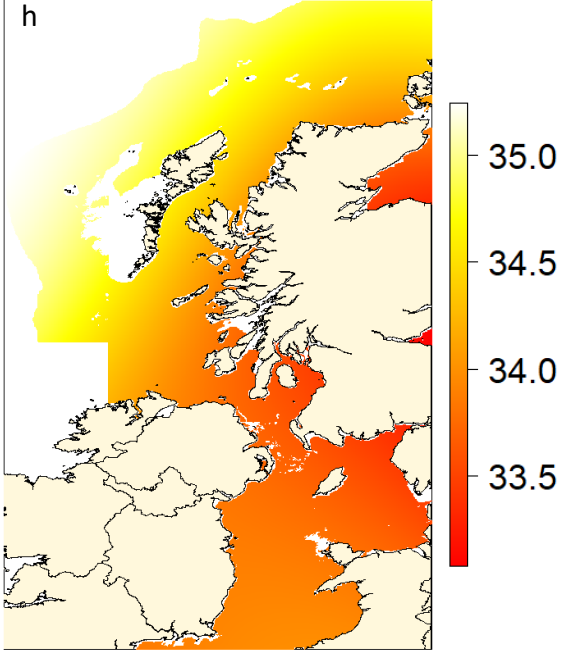

i

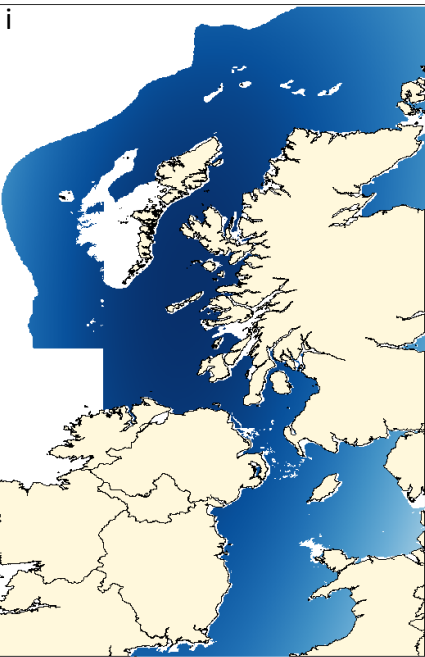

j

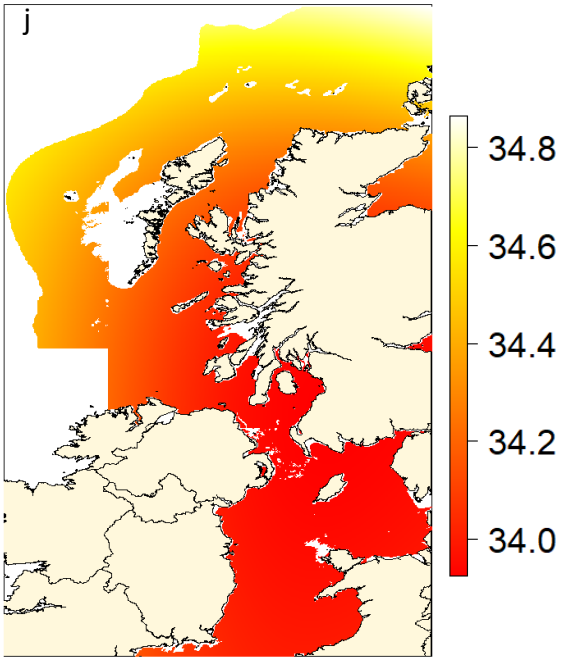

k

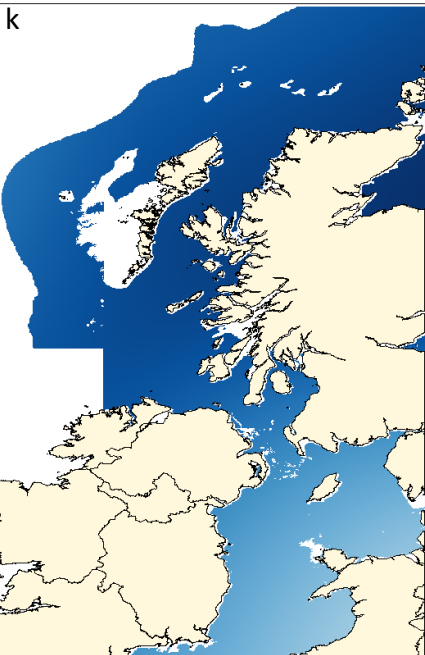

l

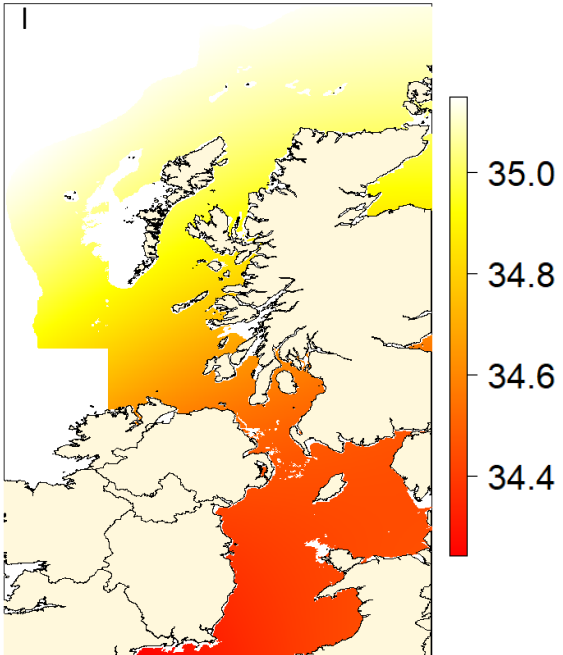

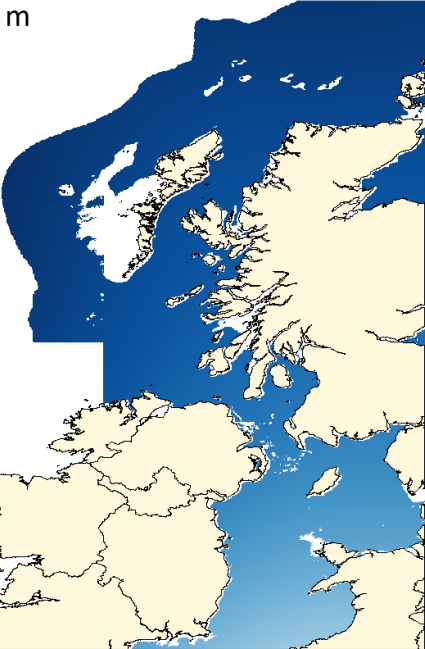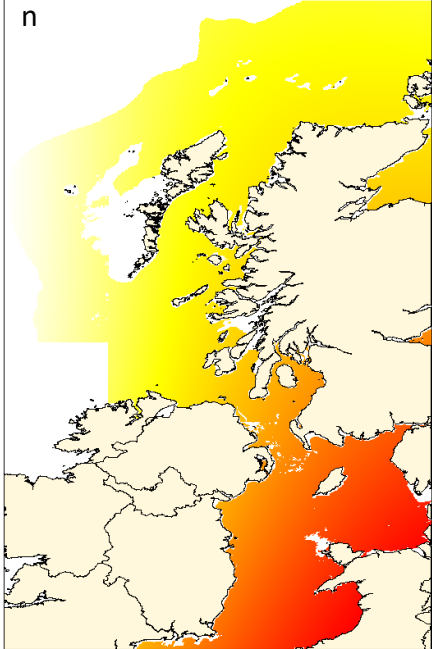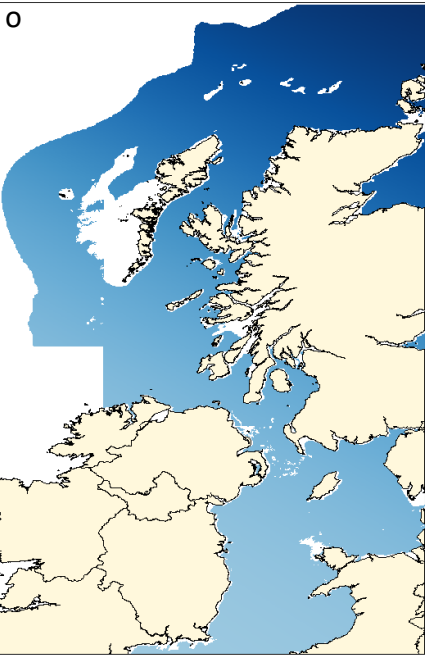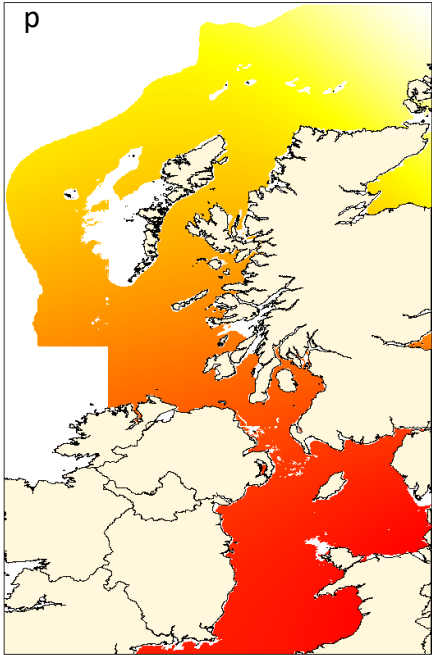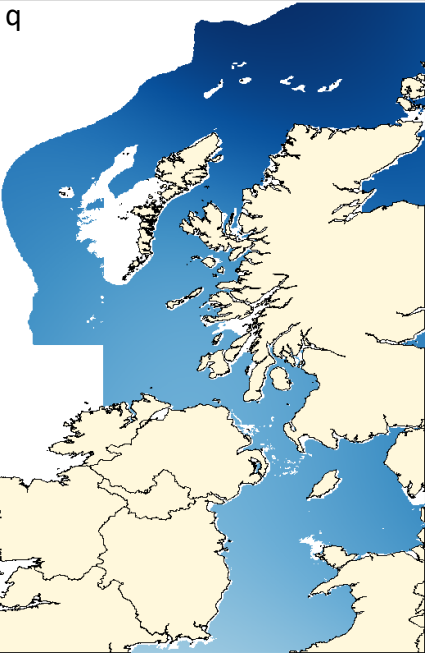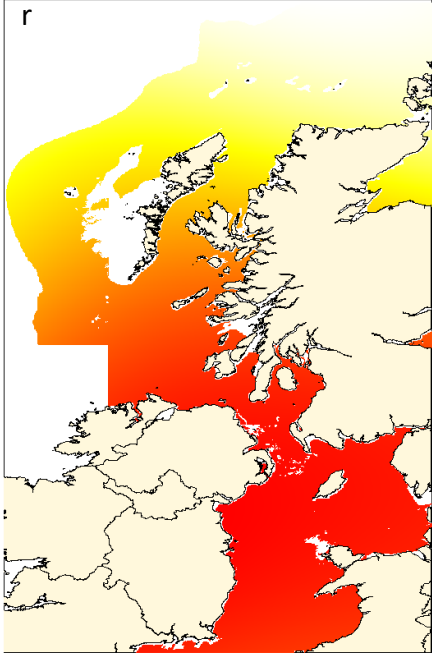

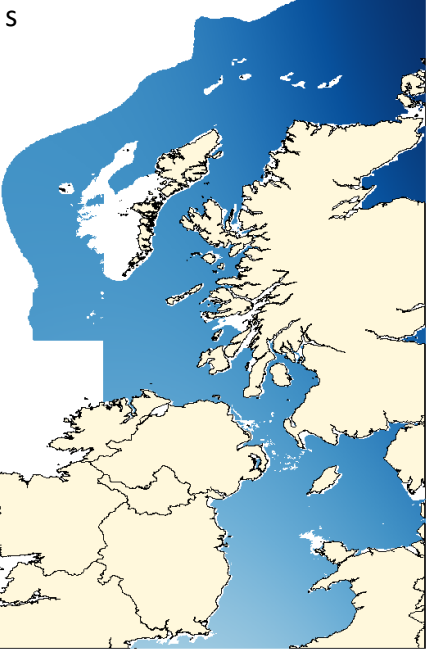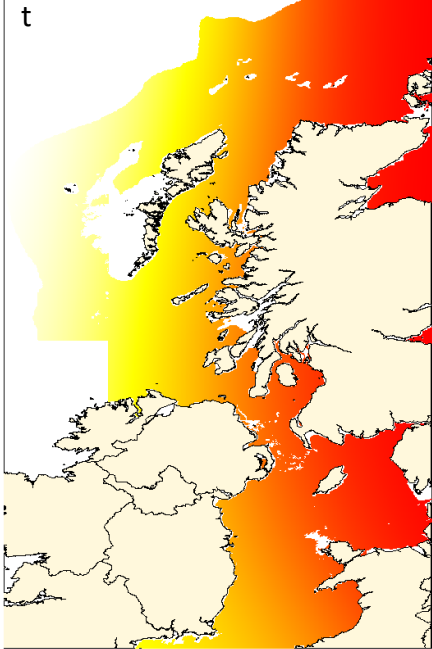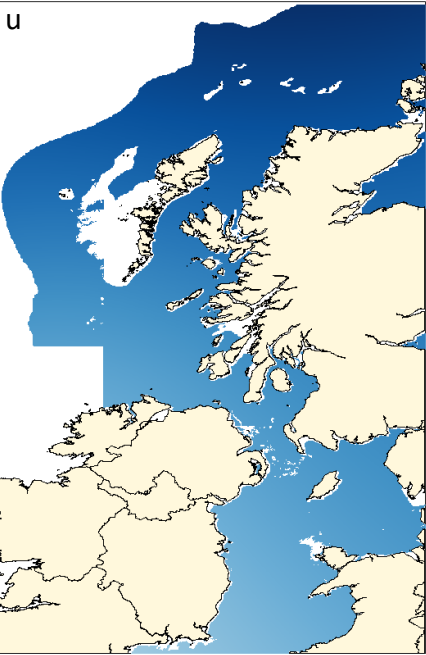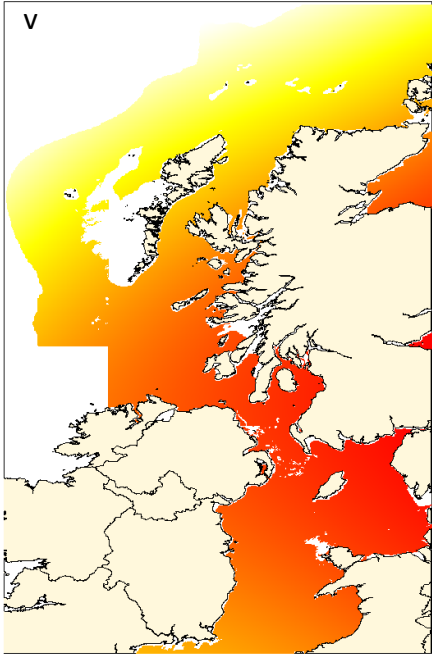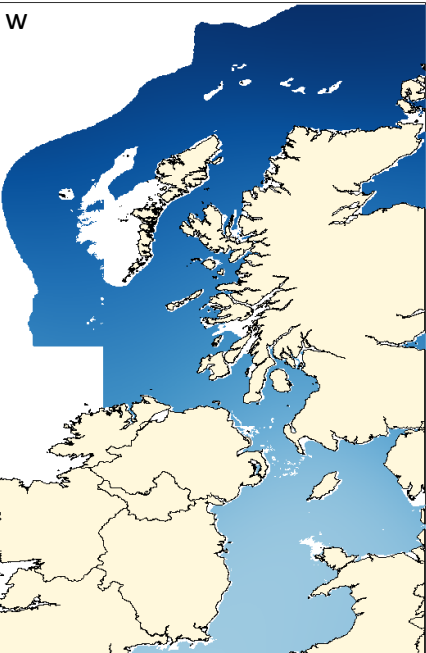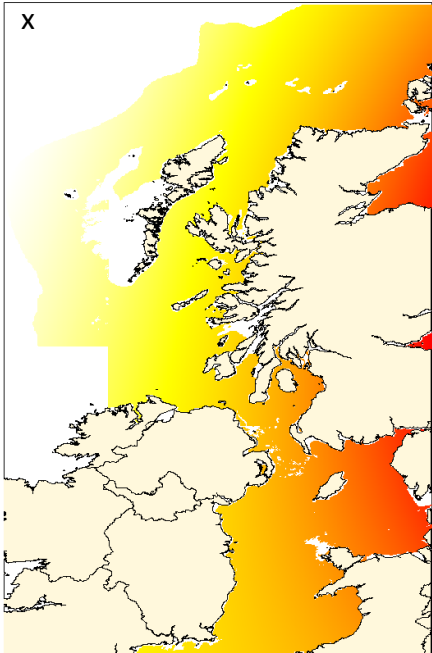

Supplement: S1 Fig — Extent of all layers dictated by the limits of available sediment data (longitude = 3°W to 10°W, latitude = 52°N to 59°N). (a) Mean bottom temperature Feb-March 2010 (°C). (b) Mean bottom salinity Feb-March 2010. (c) Mean bottom temperature Feb-March 2011 (°C). (d) Mean bottom salinity Feb-March 2011. (e) Mean bottom temperature Feb-March 2012 (°C). (f) Mean bottom salinity Feb-March 2012. (g) Mean bottom temperature Feb-March 2013 (°C). (h) Mean bottom salinity Feb-March 2013. (i) Mean bottom temperature Feb-March 2014 (°C). (j) Mean bottom salinity Feb-March 2014. (k) Mean bottom temperature Feb-March 2015 (°C). (l) Mean bottom salinity Feb-March 2015. (m) Mean bottom temperature Oct-Nov 2009 (°C). (n) Mean bottom salinity Oct-Nov 2009. (o) Mean bottom temperature Oct-Nov 2010 (°C). (p) Mean bottom salinity Oct-Nov 2010. (q) Mean bottom temperature Oct-Nov 2011 (°C). (r) Mean bottom salinity Oct-Nov 2012. (s) Mean bottom temperature Oct-Nov 2013 (°C). (t) Mean bottom salinity Oct-Nov 2013. (u) Mean bottom temperature Oct-Nov 2014 (°C). (v) Mean bottom salinity Oct-Nov 2014. (w) Mean bottom temperature Oct-Nov 2015 (°C). (x) Mean bottom salinity Oct-Nov 2015. Contains OS data Crown copyright and database right 2018 and the GEBCO_2014 Grid, version 20150318, www.gebco.net. (PDF) [file pone.0214459.s001.pdf]

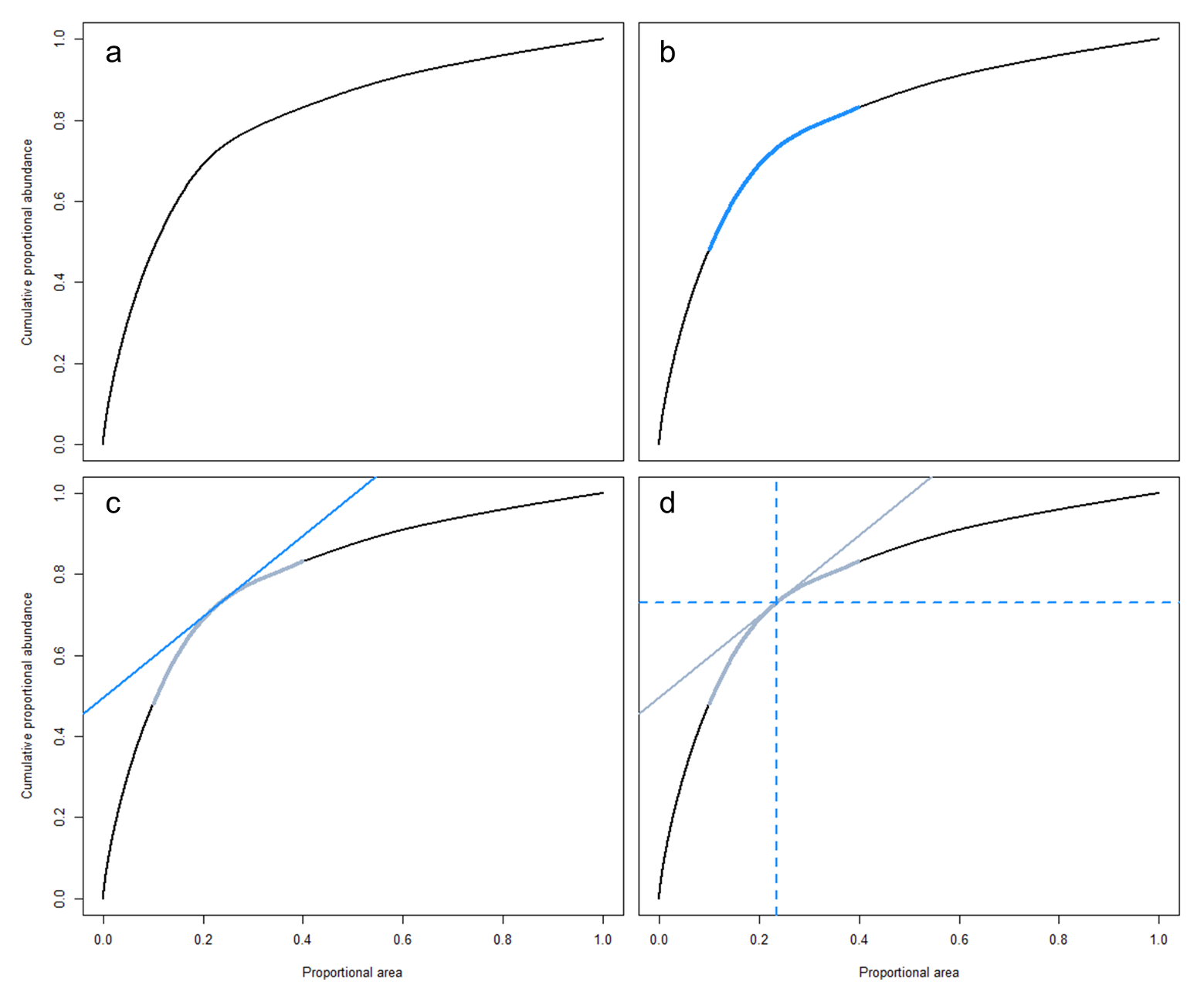

Supplement: S2 Fig — (a) Example aggregation curve plotting cumulative proportional abundance against the proportional area occupied. (b) Cubic polynomial fitted to the transition phase of the curve. (c) Derived tangent to the curve with a slope of 1 identifying the point at which space use changes from aggregated to dispersed. (d) x and y coordinates of the point where the tangent meets the curve. Y indicates the aggregated proportion of the fish distribution and thus the percentile “cut-off” to apply to the overall density distribution while x shows the proportion of total space occupied by these fish. (TIF) [file pone.0214459.s002.tif]

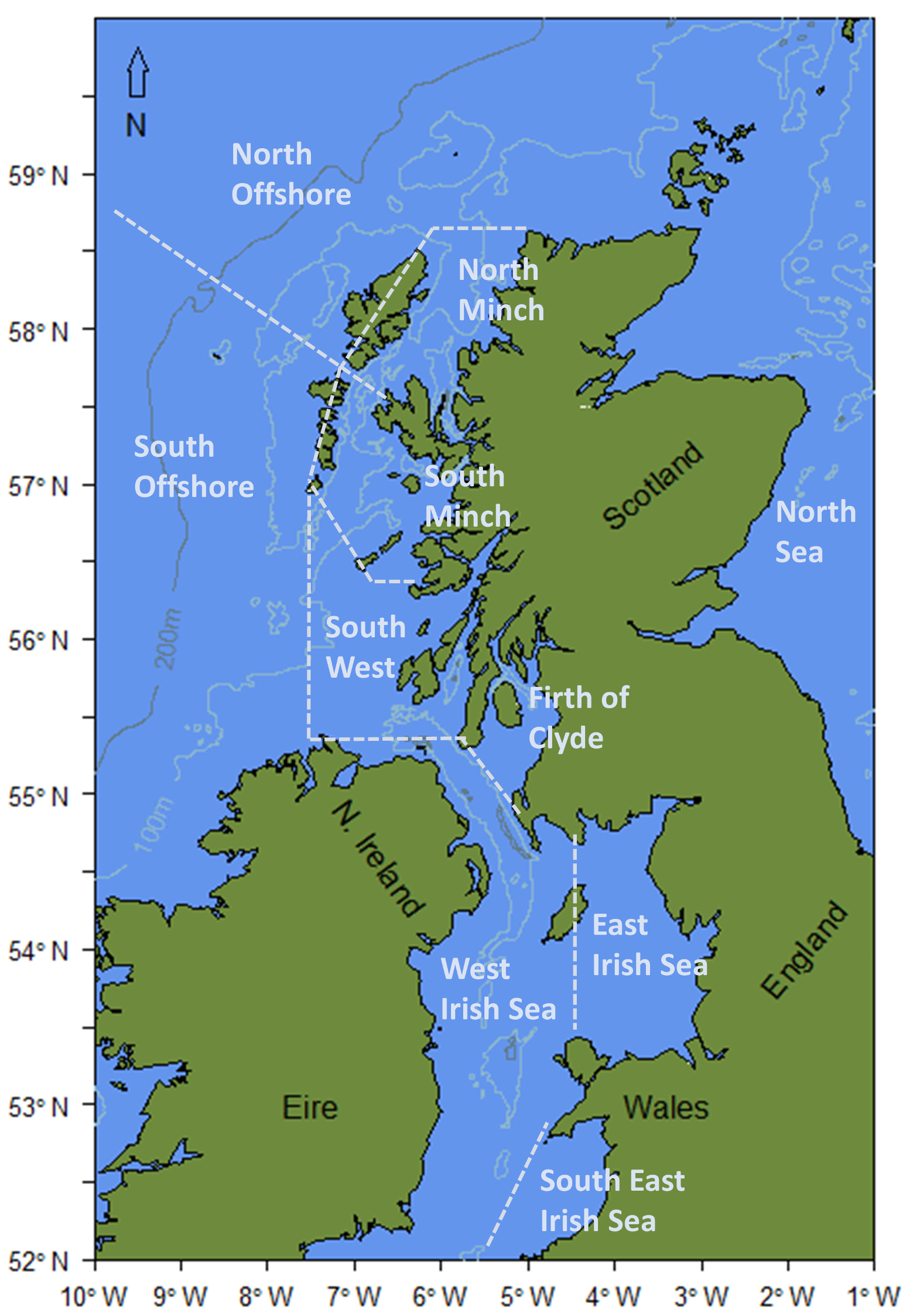

Supplement: S3 Fig — Dotted lines indicate the boundaries defining regions used in the analyses. Contains OS data Crown copyright and database right 2018 and the GEBCO_2014 Grid, version 20150318, www.gebco.net. (TIF) [file pone.0214459.s003.tif]

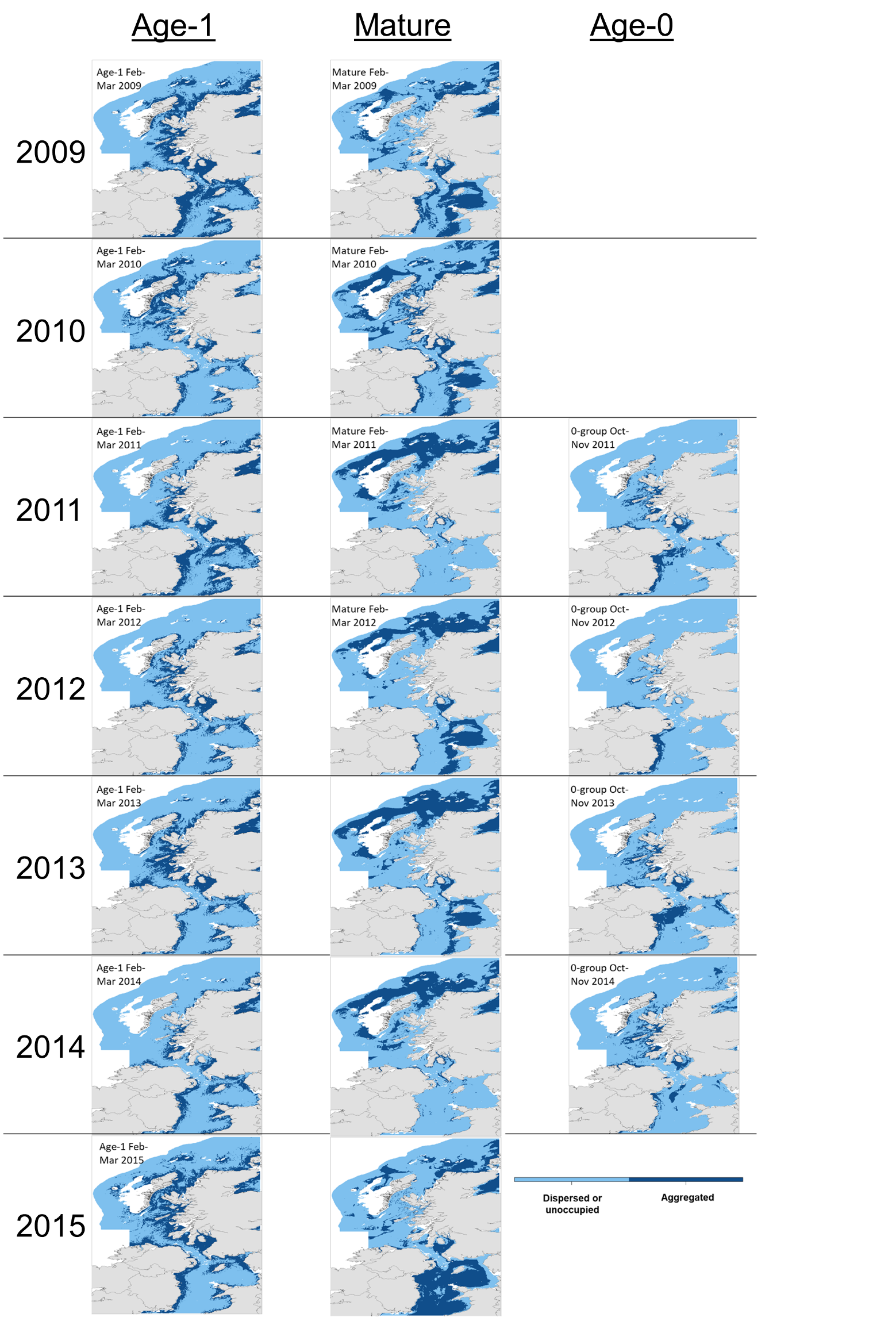

Supplement: S4 Fig — Dark blue areas delineate aggregations as defined by the threshold value derived from geostatistical aggregation curves. Contains OS data Crown copyright and database right 2018 and the GEBCO_2014 Grid, version 20150318, www.gebco.net. (TIF) [file pone.0214459.s004.tif]
