# Supplementary material for: A method to improve fishing selectivity through age targeted fishing using life stage distribution modelling
Source: PLoS One. 2019 Apr 2;14(4):e0214459. doi: 10.1371/journal.pone.0214459 (PMC6445474; doi:10.1371/journal.pone.0214459)
Supplement: S1 Table — Age-0 were modelled from October—November surveys and show the length at which the probability a fish being age-0 = 0.5. Below this length, the probability of a fish belonging to this group increases. Age-1 and mature models were constructed from surveys conducted between February and March. The probability of fish being age-1 increases for values below L50. Conversely, mature fish above L50 are more likely to be mature. (PDF) [file pone.0214459.s005.pdf]

S1 Table. Whiting length- and maturity-at-age (L_{50}, cm).

	N-Offshore		S-Offshore		N-Minch		S-Minch		S-West		Clyde		W-Irish Sea		E-Irish Sea		SE Irish Sea	
**LA_{50} age-0**	**M**	**F**	**M**	**F**	**M**	**F**	**M**	**F**	**M**	**F**	**M**	**F**	**M**	**F**	**M**	**F**	**M**	**F**
2011	23.1	25.4	22.8	24.8	23.5	25.6	23.2	24.8	22.2	23.7	22.1	23.5	19.8	20.4	17.2	17.4	19.7	20.6
2012	18.6	19.4	19.2	19.8	19.8	20.4	20.5	20.9	19.4	19.8	19.5	19.9	19.2	19.8	16.8	17.1	19.2	20.1
2013	22.7	25.5	22.9	25.4	23.0	25.8	22.7	25.4	22.5	25.2	22.5	25.1	22.3	23.1	23.5	23.9	22.5	23.5
2014	22.9	24.1	22.4	23.1	23.9	24.8	23.1	23.6	21.4	21.8	21.3	21.7	20.2	20.0	19.3	19.4	18.0	19.3
2015	22.2	22.8	22.0	22.5	23.0	23.6	22.7	23.0	21.3	21.6	21.2	21.5	19.5	19.9	19.5	19.6	19.3	20.1
Mean	21.9	23.5	21.9	23.2	22.7	24.1	22.5	23.6	21.4	22.5	21.4	22.4	20.2	20.8	19.3	19.5	19.8	20.7
± 1se	±0.8	±1.1	±0.7	±1.0	±0.7	±1.0	±0.5	±0.8	±0.5	±0.9	±0.5	±0.9	±0.5	±0.6	±1.2	±1.2	±0.7	±0.7
**LA_{50} age-1**																		
2009	22.1	22.7	21.8	22.5	21.2	21.9	21.7	22.2	22.2	22.9	21.0	21.6	20.9	21.5	20.7	21.3	20.5	21.2
2010	22.7	23.7	26.1	27.4	26.8	28.0	27.2	28.0	25.3	26.4	19.8	20.8	20.3	21.0	19.0	19.6	19.7	20.3
2011	20.5	21.1	18.7	19.7	19.1	20.0	18.4	18.9	19.6	20.4	18.7	19.4	20.2	20.8	19.3	19.9	21.2	21.9
2012	22.7	23.5	24.1	25.3	24.5	25.5	25.5	26.1	24.8	25.8	21.8	22.6	21.3	21.9	19.6	20.2	20.1	20.7
2013	21.6	22.2	21.8	22.7	23.3	24.1	24.3	24.9	22.2	22.9	20.8	21.4	19.5	20.0	19.3	19.9	20.2	20.7
2014	22.6	23.1	23.7	24.3	24.4	24.9	25.3	25.7	22.8	23.4	23.0	23.5	21.9	22.4	21.1	21.6	29.6	30.1
2015	24.0	24.9	25.3	26.6	26.2	27.3	29.9	30.6	22.4	23.5	21.0	21.8	21.1	21.5	20.6	21.0	20.2	20.5
Mean	22.3	23.0	23.1	24.1	23.7	24.5	24.6	25.2	22.8	23.6	20.9	21.6	20.8	21.3	20.0	20.5	21.7	22.2
± 1se	±0.4	±0.5	±0.9	±1.0	±1.0	±1.1	±1.4	±1.4	±0.7	±0.8	±0.5	±0.5	±0.3	±0.3	±0.3	±0.3	±1.3	±1.3
**L50 Mature**																		
2009	15.4	20.8	13.8	20.1	16.4	20.7	17.3	21.9	15.9	21.8	15.8	21.9	14.3	20.3	14.5	20.3	14.1	20.1
2010	18.4	23.3	18.4	23.9	19.5	23.3	17.7	22.2	16.7	22.4	15.8	19.9	15.9	20.1	15.6	19.9	15.6	19.9
2011	13.2	19.1	13.2	19.6	14.4	19.2	16.0	20.8	12.1	18.7	14.2	18.5	14.4	18.9	15.9	19.9	14.3	18.8
2012	16.8	22.0	16.4	22.3	19.3	23.2	19.4	23.7	17.1	22.8	15.5	19.6	16.2	19.6	15.8	19.4	15.6	19.2
2013	14.5	20.2	16.2	22.1	16.9	21.2	18.3	22.7	14.8	21.0	16.9	20.7	14.6	19.2	15.1	19.5	16.2	20.4
2014	18.6	23.4	18.5	24.0	17.6	21.7	21.8	25.5	18.1	23.6	19.0	22.5	19.3	21.9	17.9	21.0	29.0	32.2
2015	19.2	24.0	17.3	23.0	19.7	23.4	21.3	25.2	17.4	23.0	14.9	19.1	16.8	20.9	15.2	19.8	16.2	20.5
Mean	16.6	21.9	16.3	22.2	17.7	21.9	18.9	23.2	16.0	22.0	16.0	20.4	15.9	20.2	15.7	20.0	20.2	23.1
± 1se	±0.9	±0.7	±0.8	±0.7	±0.7	±0.6	±0.8	±0.7	±0.8	±0.6	±0.6	±0.6	±0.7	±0.4	±0.4	±0.2	±2.0	±0.4

Age-0 were modelled from October - November surveys and show the length at which the probability a fish being age-0 = 0.5. Below this length, the probability of a fish belonging to this group increases. Age-1 and mature models were constructed from surveys conducted between February and March. Probability of fish being age-1 increases for values below L_{50} and above L_{50} fish are increasingly likely to be mature.
